# Plant communities in harsh sites are less invaded: a summary of observations and proposed explanations

**DOI:** 10.1093/aobpla/plv056

**Published:** 2015-05-22

**Authors:** Emily Zefferman, Jens T. Stevens, Grace K. Charles, Mila Dunbar-Irwin, Taraneh Emam, Stephen Fick, Laura V. Morales, Kristina M. Wolf, Derek J. N. Young, Truman P. Young

**Affiliations:** 1 Department of Plant Sciences, University of California, Davis, CA 95616, USA; 2 John Muir Institute for the Environment, University of California, Davis, CA 95616, USA; 3 Present address: Department of Earth and Planetary Sciences, University of Tennessee, Knoxville, TN 37917, USA

**Keywords:** Environmental stress, invasibility, invasive/exotic plants, native plant refuges, propagule pressure, resource availability

## Abstract

Plant communities in abiotically stressful, or ‘harsh’, habitats have been reported to be less invaded by non-native species than those in more moderate habitats. Here, we synthesize descriptive and experimental evidence for low levels of invasion in habitats characterized by a variety of environmental stressors: low nitrogen; low phosphorus; saline, sodic or alkaline soils; serpentine soils; low soil moisture; shallow/rocky soils; temporary inundation; high shade; high elevation; and high latitude. We then discuss major categories of hypotheses to explain this pattern: the propagule limitation mechanism suggests invasion of harsh sites is limited by relatively low arrival rates of propagules compared with more moderate habitats, while invasion resistance mechanisms suggest that harsh habitats are inherently less invasible due to stressful abiotic conditions and/or increased effects of biotic resistance from resident organisms. Both propagule limitation and invasion resistance may simultaneously contribute to low invadedness of harsh sites, but the management implications of these mechanisms differ. If propagule limitation is more important, managers should focus on reducing the likelihood of propagule introductions. If invasion resistance mechanisms are in play, managers should focus on restoring or maintaining harsh conditions at a site to reduce invasibility.

## Abstract (precis)

Within the invasion ecology literature, it is often noted that abiotically stressful environments are typically less invaded by non-native plants than nearby less-stressful environments. However, until now no one had collected and summarized examples of this pattern. This paper first compiles evidence that plant communities in many harsh habitats are less invaded, and then synthesizes possible explanations for this pattern. We discuss that harsh sites may be less invaded because, compared to moderate sites, they may receive lower propagule pressure, particularly from well-suited plants, and because their abiotic and biotic characteristics may make them inherently more resistant to invasion.

## Introduction

Discerning the patterns and underlying causes of plant invasions is a central goal of invasion ecology. Many studies have attempted to identify characteristic traits of invasive plant species and invasible communities (e.g. Rejmánek 1989; Lonsdale 1999; Alpert *et al*. 2000; Theoharides and Dukes 2007; Chytrý *et al*. 2008; Rejmánek *et al*. 2013; Gioria and Osborne 2014). In addition to identifying which habitats tend to be highly invaded—often disturbed and high-resource sites—these and other authors often note that some habitats are characteristically *less* invaded than others—specifically, abiotically stressful, or ‘harsh’, sites. However, support for this assertion is often given in the form of individual examples, and there has not yet been a detailed compilation of harsh habitats that are reported to be less invaded. Furthermore, there has not yet been a synthesis of hypotheses for why harsh habitats may be less invaded than more moderate environments. In this paper, we provide a summary of evidence for the phenomenon of low invadedness of harsh habitats and discuss hypotheses for understanding this pattern. We first compile an annotated list of harsh habitats reported to have low levels of invasion by non-native plants, and then outline two major classes of hypotheses for why harsh habitats may be less invaded—propagule limitation mechanisms and invasion resistance mechanisms—and discuss their management implications.

We define harsh habitats as sites with the regular presence of one or more abiotic stressors, which can include naturally low levels of critical plant resources (e.g. nitrogen, light, oxygen or water), presence of toxins (e.g. heavy metals or salts) or temperature extremes (Table 1). Harsh habitats share the characteristic of abiotic stress, which limits the rate of resource acquisition, growth or reproduction in resident plants (Grime 1989). To persist in harsh habitats, plants must possess resource conservation or acquisition strategies to adapt to resource scarcity (e.g. increased resource use efficiency, nutrient resorption, modified roots; Funk 2013), or tolerance/avoidance strategies to deal with chemical toxicity (e.g. synthesis of detoxifying metabolites and proteins, ion compartmentalization or exclusion; Raskin *et al*. 1994; Parida and Das 2005) or freezing (e.g. production of ‘antifreeze’ proteins; Pearce 2001).

**Table 1.** Physiological effects of stressors present in harsh habitats.

Stressor	Habitat(s) listed in this paper where stressor is present	Physiological effects on plants	References
Low nitrogen	Nitrogen-poor sites (including calcareous earth, limestone outcrops, volcanic ash); serpentine sites; bogs; rocky outcrops	Impaired protein synthesis; chlorosis; reduced leaf turgor; reduced leaf/tiller number; reduced growth rate; low seed yield	Nightingale (1948), Orcutt and Nilsen (2000), Walker (1954)
Low phosphorus	Phosphorus-poor environments; serpentine sites; bogs	Reduced seed size and root : shoot ratios; increased water stress and leuco-anthocyanin content; reduced leaf/tiller number; reduced growth rate; low seed yield	Atkinson (1973); Orcutt and Nilsen (2000); Walker (1954)
Low Ca : Mg ratio	Serpentine sites	Limited root growth and root activity; cell membrane disintegration or weak membranes; reduced uptake of other nutrients	Bangerth (1979), Kruckeberg (1954), McNaughton (1968), Walker (1954)
High salinity	Saline, sodic sites	Growth stunting and reduced fruiting/flowering; lower water availability (negative water potential in soil); osmotic and ionic imbalance; oxidative damage	Bernstein (1975), Parida and Das (2005)
High alkalinity	Sodic, alkaline sites	Fe, Mn, Zn, Cu deficiency due to cation precipitation; impaired enzyme synthesis/function; impaired root growth due to poor soil structure	Mengel *et al*. (2001), Orcutt and Nilsen (2000)
Heavy metals	Serpentine sites	Growth stunting; induced iron deficiency; chlorosis; restricted root development	Foy *et al*. (1978), Kruckeberg (1954), McNaughton (1968), Walker (1954)
Low soil moisture	Xeric sites; rocky outcrops; serpentine sites	Reduced nutrient uptake and transport; decreased stomatal opening and reduced photosynthetic capacity; reduced plant growth and productivity	Bangerth (1979), Osakabe *et al*. (2014)
Anoxia	Periodically inundated sites; bogs	Energy starvation; cell damage via ethanol buildup, cytoplasmic acidosis, free radicals; reduced nutrient uptake and transport	Vartapetian and Jackson (1997)
High acidity	Bogs	Damage to root tips; toxicity due to greater availability of metals (Al, Mn); nutrient deficiency from inhibited uptake of metal cations (K, Mg, Ca) or decreased solubility of elements (P, Mo)	Marschner (1991), Mengel *et al*. (2001)
Low light	Shaded terrestrial and aquatic environments; high latitude sites (winter season)	Reduced photosynthate availability; reduced biomass allocation to roots and reproductive structures (flower and seed); higher shoot to root ratios and investment in shoot elongation	Begna *et al*. (2002)
Freezing temperatures	High altitudes; high latitudes	Low water availability in soil; slower metabolism; freezing-induced cellular dehydration; ice-induced blockages in vessels and organs; cellular damage	Pearce (2001)
High UV-B radiation exposure	High altitudes	DNA damage; damage to photosynthetic apparatus; inhibition of photosynthesis; reduction of above and belowground growth; reduction in foliage size; altered reproductive output and timing	Caldwell *et al*. (1998), Rozema *et al*. (1997)

We consider a harsh site less invaded if the richness, cover and/or biomass of non-native invasive species is lower compared with similar, less harsh sites. When a stressor occurs in discrete patches (e.g. edaphic stressors or dense forest shade), less harsh sites can include the habitat matrix adjacent to patches of harsh habitat. When a stressor is caused by continuous climatic variation (e.g. low temperatures or aridity), less harsh sites are defined by their position along the stress gradient. Because native species richness or cover may also decline with greater abiotic stress, when available we include information on whether richness or cover of invasives in harsh sites is lower *relative* to natives. In cases where the proportion of invasive species out of the total species pool declines with increasing stress, this suggests that there is something unique about the phenomenon of invasion that causes this pattern (e.g. different traits of invasive vs. native species or differences in propagule pressure between harsh and moderate sites). Regardless of the corresponding trend in natives, differences in total invasive richness or abundance across a stress gradient provide useful information to managers trying to exclude invasive species.

Throughout this paper we use the term ‘invasives’ to mean non-native plant species capable of establishing, spreading and causing ecological and/or economic damage, and the term ‘non-natives’ to simply indicate species outside of their native range. Although we focus on invasive species, we sometimes include examples of non-natives that are not reported to be problematic because (i) evidence of impacts may not be well known or described and (ii) such species may eventually become invasive after a lag period (Simberloff 2009). We use the term ‘non-native(s)’ when there is a lack of evidence of impacts or when describing multiple non-indigenous species together, some of which may not be known invasives. We also note that hypotheses for low invadedness of harsh sites can also apply to patterns of invasion by native species (Alpert *et al*. 2000), and discuss certain cases where relevant.

After compiling a list of examples, we discuss two major classes of hypotheses that have been proposed to explain why harsh habitats are less invaded. (i) *Propagule limitation mechanisms* suggest that characteristics of harsh sites, such as isolation, small size and lower rates of human visitation and disturbance, may reduce their exposure to non-native propagules, which in turn limits successful invasions (Lockwood *et al*. 2005; Simberloff 2009). (ii) *Invasion resistance mechanisms* invoke the stressful conditions of harsh habitats as either direct or indirect causes of reduced invasion (Alpert *et al*. 2000; Shea and Chesson 2002). Specifically, the pool of potential invaders may either be physiologically intolerant of the stressful conditions, and/or the stressful conditions might increase the impacts of biotic resistance from resident native species. These two classes of hypotheses are not mutually exclusive, and in many cases both may be in effect. While we do not seek to test the relative importance of these hypotheses in particular harsh habitats or overall, we note that their implications for management of plant invasions in harsh habitats may differ.

## Evidence that Harsh Habitats Are Less Invaded

For each class of harsh habitat listed below, we provide examples of published research that give observational evidence of low invadedness in these sites, and where available, experimental evidence that invasion success or competition between natives and invasives can be altered by manipulations of the proposed stressors. We also present counter-examples where relevant. A comprehensive review of all relevant examples or a meta-analysis is beyond the scope of this review. The literature reviewed here is summarized in the **Supporting Information**.

### Nitrogen-poor sites

Many natural ecosystems are relatively low in available soil nitrogen (N), and N is often a limiting factor for plant growth (Vitousek and Howarth 1991). Naturally low N levels may contribute to why some terrestrial ecosystems, such as calcareous and sandy grasslands (Kolb *et al*. 2002) and arid and semi-arid communities (McLendon and Redente 1991; Brooks 2003), have low cover of non-natives. Inversely, areas of higher fertility in relatively low-nutrient systems such as alpine communities have a higher mean number of non-native species, compared with lower fertility areas (McDougall *et al*. 2005). Higher N availability is linked with higher rates of invasion generally: the frequently observed pattern of disturbance favouring invasive species over natives has often been attributed to increased availability of N after disturbance (Hobbs *et al*. 1988; reviewed in Davis *et al*. 2000).

Experimental studies have shown that increases in soil N tend to benefit invasive species more than native species in a variety of terrestrial systems, including both those severely and moderately poor in nitrogen (Wedin and Tilman 1996; Kolb *et al*. 2002; Abraham *et al*. 2009; Liancourt *et al*. 2009; see also review by James *et al*. 2011), implying reduced success of invasives relative to natives in N-poor sites. Experiments have shown similar patterns in aquatic systems: compared with native macrophytes (aquatic plants), non-native submersed macrophyte biomass (Chase and Knight 2006) and asexual propagule performance (Xie *et al*. 2010) were greater in high N treatments. Greater relative performance of non-natives over natives in N-enriched treatments has been found in wetland plants as well (Rickey and Anderson 2004; Holdredge *et al*. 2010). Moreover, anthropogenic N enrichment in the form of agricultural activities or roadside pollutants increase biomass and richness of N-loving invasive species over native species in low-N plant communities such as calcareous grasslands in the Netherlands (Willems 2001; Lee and Power 2013), sandy grasslands of Hungary (Török *et al*. 2014), coastal grasslands in California (Kolb *et al*. 2002) and arid (Brooks 2003) and semi-arid ecosystems in western North America (McLendon and Redente 1991). Similarly, Maron and Connors (1996) concluded that the presence of an N-fixing shrub species facilitates greater cover by non-native species in a California coastal prairie at the expense of the number and cover of native species.

Additional evidence that low nitrogen levels hinder invasion is that intentional N impoverishment carried out by restoration practitioners, especially in grassland systems, can reduce the impact of invasive plants, again not always in the context of systems that are considered particularly poor in nitrogen (Morgan 1994; Paschke *et al*. 2000; Prober *et al*. 2005; Steers *et al*. 2011; Török *et al*. 2014; reviewed by James *et al*. 2011). Reducing soil N can increase abundance of native species relative to invasive (or ‘weedy’) species, although success has been mixed (Wilson and Gerry 1995; Burke *et al*. 2013; also reviewed by Corbin *et al*. 2004; James *et al*. 2011). Techniques for N-impoverishment include biomass removal (Willems 2001; Perry *et al*. 2010), topsoil removal (Buisson *et al*. 2008) and carbon addition (Wilson and Gerry 1995; Reever Morghan and Seastedt 1999; Burke *et al*. 2013).

### Phosphorus-poor environments

Phosphorus (P) limitation may be a barrier to invasion, though it is often confounded with co-limitation of other nutrients (Kueffer *et al*. 2008; Haubensak and D'Antonio 2011). Numerous studies have found a positive relationship between soil P levels and both richness and cover of non-native species, and a negative relationship between native species richness and increased P. For example, P-enrichment in naturally low-P Australian urban bushland was associated with higher non-native richness and lower native richness (Lake and Leishman 2004). Morgan (1998) found much higher soil P levels at the invaded edges of a *Themeda trianda* remnant Australian grassland than in the less invaded interior. In observational studies in Australian Banksia woodland (Fisher *et al*. 2006), subtropical wetland (Boughton *et al*. 2011) and riparian zones in southeast China (Xiao-Yun *et al*. 2006), high-P environments were more invaded than low-P environments. Dimitrakopoulos *et al*. (2005) found that soil P was positively correlated with aboveground biomass of non-native species in a Mediterranean grassland.

Experimental evidence has shown that increased P is associated with greater success of invasive species relative to natives in both terrestrial and aquatic systems. As with soil N, experimentally increasing soil P tends to lead to superior performance of invasives relative to natives. For example, in a greenhouse experiment Leishman and Thomson (2005) found that when nutrients were added to low P soils in Hawkesbury Sandstone communities in Australia, invasive species had higher survival and growth rates than natives. Leishman *et al*. (2004) found that increased soil P in stormwater runoff areas in Australia (defined as >150 mg kg^−1^) resulted in significantly increased invasive species cover proportional to natives. Cherwin *et al*. (2009) found that both absolute and relative cover of invasive grasses (*Bromus* spp.) in experimental plots in a Colorado grassland were positively associated with P addition. In serpentine soils, P fertilization increased the invasion rate and dominance of invasives within 2 years (Huenneke *et al*. 1990).

Phosphorus is often the most limiting nutrient in freshwater aquatic systems, and enrichment of waters with P and other nutrients is thought to increase invasibility (Engelhardt 2011). For example, invasive *Hydrilla verticillata* (Royle) (hydrilla) presence was positively correlated with total P in Florida lakes (Gu 2006), and hydrilla had a competitive advantage over native *Vallisneria americana* (American eelgrass) in higher P (and other nutrient) soils, while eelgrass was the stronger competitor in nutrient-limited soils (Van *et al*. 1999).

### Saline, sodic or alkaline environments

Environments with highly saline, sodic and/or alkaline soils, such as tidal or inland salt marshes and alkali sinks, have been anecdotally observed to be less invaded (Baker 1986). Salinity, sodicity and alkalinity can negatively affect plant growth through ion toxicity, effects on osmotic potential and interference with plant nutrition. These sites are also often temporarily inundated, which can subject plants to anoxia.

Though wetlands are generally susceptible to invasion (Zedler and Kercher 2004), cover of non-natives decreased along a gradient of increasing salinity in Southern California salt marshes, while native cover increased with salinity (Uyeda *et al*. 2013). Other studies have found that particular wetland invaders are unable to establish in highly saline areas (e.g. Zedler *et al*. 1990; He *et al*. 2012; but see Daehler and Strong 1996). Human alteration of hydrological processes through shoreline development and water management practices (e.g. controlled flooding) can decrease salinity and facilitate invasion by non-native brackish species (Zedler *et al*. 1990; Mesléard *et al*. 1993; Konisky and Burdick 2004; Silliman and Bertness 2004). Application of salt in these areas has been proposed as a method for controlling non-natives without harming the native plant community (Kuhn and Zedler 1997; Uyeda *et al*. 2013). Conversely, increased salinity has been implicated in the success of invasive salt-tolerant tamarisk (Hultine *et al*. 2010).

Research on invasion of sodic or alkali soils is sparse, though it appears that these sites can act as edaphic refuges for native species (Dawson *et al*. 2007). For example, non-native *Lolium multiflorum* (ryegrass; now *Festuca perennis*) that dominated a non-sodic matrix was virtually absent in adjacent alkali sinks, whereas native *Hemizonia pungens* ssp. *pungens* was more abundant in the alkali sinks (Veblen and Young 2009). It is likely that amelioration of naturally sodic soils for the purpose of agriculture (for example, by addition of gypsum: see Qadir *et al*. 2001) may displace native species and facilitate invaders, though experimental evidence for this is currently lacking.

### Serpentine sites

Serpentine soils are edaphically harsh, often characterized by low macronutrient (N, P, S) and micronutrient (Ca) content, high concentrations of toxic heavy metals (Cr, Ni), low Ca : Mg ratio and low soil moisture (Whittaker 1954; Chiarucci and Baker 2007). While western North American grasslands are highly invaded relative to other habitat types (e.g. forests), interspersed serpentine ‘islands’ have been historically less invaded relative to surrounding non-serpentine grasslands (Kruckeberg 1954; Walker 1954; Baker 1986). Many native species that were widespread prior to invasion by Mediterranean annuals in California's grasslands find refuge in serpentine soils (Kruckeberg 1984; Huenneke *et al*. 1990), where natives are generally more abundant than on non-serpentine soils, either in absolute terms or relative to non-natives (McNaughton 1968; Harrison 1999*a*). In northern California's Sedgewick Reserve, native species richness was higher than invasive species richness on spatially isolated rocky serpentine outcrops/hummocks, while the converse was true in the surrounding serpentine grassland. This was attributed to abiotically stressful conditions on shallow, lower-nutrient hummocks (Gram *et al*. 2004). Within serpentine sites in a northern California grassland, species diversity of invasives relative to natives increased with increasing P, Ca : Mg ratios, soil depth and water-holding capacity. Moreover, there was higher absolute and relative native species richness in serpentine than non-serpentine meadows (Harrison 1999*b*). Serpentine plant communities in New Caledonia are similarly depauperate in non-natives (Jaffre 1992).

Experimental manipulations have shown that amelioration of serpentine stressors tends to favour invasive species over natives. In California, nutrient addition to serpentine soils generally increased biomass and fecundity of invasive grasses to a greater extent than native species (Huenneke *et al*. 1990; O'Dell and Claassen 2006; Going *et al*. 2009). Hobbs *et al*. (1988) found that invasive grasses almost entirely replaced native forbs in fertilized serpentine plots. These studies, along with the observations that anthropogenic N deposition is linked to greater invasion of serpentine sites (Turitzin 1982; Weiss 1999, 2006), suggest that N limitation may be the most important stressor controlling invasion in these systems.

### Xeric sites

Water availability often limits plant growth and acquisition of other resources in terrestrial environments (Chapin *et al*. 1987). Across soil moisture gradients, xeric locations generally tend to have a lower proportion of invaders than mesic locations (Cronk and Fuller 1995; Rejmánek *et al*. 2013). This pattern is evident in a wide variety of systems, including temperate forests (Rejmánek *et al*. 2013), Hawaiian grasslands (Goergen and Daehler 2001), temperate grasslands (Larson *et al*. 2001), semi-arid savannahs (Harrington 1991) and deserts (Bashkin *et al*. 2003; Miller *et al*. 2006; Brooks 2009). When arid landscapes contain patches with greater soil moisture, these patches are often more highly invaded (e.g. Stohlgren *et al*. 2001). The importance of moisture in determining patterns of invasion is also evident from temporal fluctuations in water availability; for example, wet years tend to favour non-natives in arid and semi-arid systems (Burgess *et al*. 1991; Hobbs and Mooney 1991; Dyer and Rice 1999). In contrast, however, Lortie and Cushman (2007) showed that the proportion of exotic species increased along a natural coastal dune gradient of decreasing soil moisture (and nitrogen).

Experimental evidence suggests that soil moisture may influence the relative performance of native and invasive species. Daehler (2003) found natives to have superior physiological performance compared with invasives under water-limited conditions in 8 of 12 studies reviewed. Similarly, water additions favoured non-natives over natives in a New Zealand grassland (White *et al*. 1997) and in shortgrass steppe (Milchunas and Lauenroth 1995).

However, while there appears to be a general trend of invaders responding positively to water addition, this is not always the case. Seabloom *et al*. (2003) found that year-round water additions did not significantly increase invasion of non-native annual grasses into native perennial stands in a California grassland, but instead enhanced native perennial grass reestablishment within stands of non-native annuals. Similarly, water additions had no significant impact on invasion or competitive interactions between several noxious invasives and monocultures of 10 native perennials in a water-limited system in western Montana (Maron and Marler 2008) or invasion of *Holcus lanatus* into perennial grass stands in California coastal grasslands (Thomsen and D'Antonio 2007).

There is also some question as to whether the pattern of lower richness of invasive species at drier sites holds with respect to cover of invasive species. Historically, deserts have been considered more resistant to invasion than more mesic biomes due to perceived physiological stress of chronic water limitation (Baker 1986; Loope *et al*. 1988). In a global analysis of plant species data from nature reserves, Lonsdale (1999) found that sites in deserts and savannas had proportionally fewer non-native species than those in more mesic biomes. However, many arid and semi-arid rangelands are now highly invaded, often by a small number of species. For example, *Bromus tectorum* (cheatgrass) has become the dominant weed of the US Great Basin (Knapp 1996) and is able to invade even isolated and undisturbed locations (Belnap and Phillips 2001). Other examples from deserts include *Brassica tournefortii* (Asian mustard) and *Pennisetum ciliare* (buffelgrass) (Rejmánek *et al*. 2013). Thus, if well-adapted non-natives can reach arid sites, they may become successful invaders.

### Rocky outcrops and shallow soils

In rocky and/or shallow soils, patterns of invasion may be affected by a number of stressful conditions, including reduced access to water and nutrients, extremes in soil temperatures and mechanical resistance to soil disturbance (Houle and Phillips 1989). Soil depth influences plant community dynamics (Grime and Curtis 1976), and shallow soils often have lower invasive cover relative to natives than adjacent deep soils (Kolb *et al*. 2002; Talluto and Suding 2008; Emam 2015; but see MacDougall *et al*. 2006). Similarly, rocky outcrops often have a lower proportion of invasive species than the surrounding matrix, such as in non-native dominated coastal scrub grasslands of California (Steers 2011), piedmont of the southeastern USA (Wyatt 1997), South African inselbergs (Hopper 2002) and shallow-soil sites in New Zealand (Wiser and Buxton 2008). However, some shallow-soil sites in Australia (Hopper 2002) and tropical inselbergs in parts of Africa and Latin America (Porembski 2000) are highly invaded, at least in terms of absolute numbers of species. Invasion in these contexts may be due to increased human disturbance and/or higher fertility of interstitial soils compared with other shallow-soil rocky outcrops (Porembski 2000; Hopper 2002). The latter hypothesis suggests that nutrient limitation may drive invasion patterns in these sites.

### Periodically inundated environments

Terrestrial systems that flood on a seasonal or periodic basis, such as vernal pools, ephemeral riparian areas and wetland margins, are characterized by another form of harshness: plants that inhabit these systems must tolerate or avoid periods of anoxia. Many plants that are invasive in upland habitats are intolerant of prolonged submersion and are therefore unable to establish permanently in adjacent inundation-prone areas. Vernal pools in the Central Valley of California, for example, are often regarded as ‘islands’ of native plant communities within a matrix of heavily invaded grassland (Holland and Jain 1988). The deepest (most heavily inundated) pools generally have the lowest richness of invasives and the lowest cover relative to natives (Gerhardt and Collinge 2003), likely due to reduced growth and reproduction of invasives when inundated (Gerhardt and Collinge 2007).

Similarly, although wetlands and riparian areas are generally highly invaded (Zedler and Kercher 2004), some periodically inundated riparian areas and wetland margins are less invaded than surrounding uplands. Evidence for reduced performance of non-natives with increasing inundation intensity occurs in both absolute terms (e.g. Stokes 2008; Tanentzap *et al*. 2014) and relative to natives (e.g. Mills *et al*. 2009). Wetlands that are substantially less invaded than surrounding uplands may experience particularly harsh inundation. Indeed, a study comparing the invasibility of riparian wetlands before and after implementation of flow control (amelioration of inundation intensity) found that when inundation became less intense, cover of non-native species increased, both in absolute terms and relative to natives (Catford *et al*. 2011). Further, in a vernal pool mesocosm experiment, deeper pools—which experienced more intense inundation—were more resistant to invasion (Collinge *et al*. 2011). Similarly, Tanentzap *et al*. (2014) found that ephemeral wetland plant communities that experienced more intense flooding contained a smaller proportion of non-native species compared with native species. Comparable dynamics exist in rice fields, and rice farmers have long employed periodic flooding to control (primarily non-native) weeds (Smith and Fox 1973; Williams *et al*. 1990).

Despite their harshness, periodically inundated areas may in some cases be prone to invasion. Although inundation may generally displace invasive species more readily than natives, it can also displace natives (e.g. Collinge *et al*. 2011) and increase availability of space, light and other resources, thereby ameliorating other forms of stress (e.g. dry, low-light or low-nutrient conditions) linked with resistance to invasion. In studies finding long-term dominance of invasives in inundated areas, the success of invasives may be due to a superior ability to colonize disturbed, post-inundation habitats (Barden 1987; Collinge *et al*. 2011) or a superior ability to compete under either inundated or non-inundated conditions (Taylor and Ganf 2005; Schooler *et al*. 2010). Some work suggests that despite being invasible, inundated areas may exhibit a pattern of pulsed ‘resetting’ of invasion each time an area floods (Flores *et al*. 2005; Stokes 2008; Mills *et al*. 2009), implying a dynamic equilibrium of partial invasion that may persist indefinitely.

### Bogs

Bogs receive water mainly from precipitation, are characteristically low in nutrients and oxygen and are highly acidic. These stressors may limit invasibility to non-specialized plants, either singly or in combination. While most other types of freshwater wetlands are highly invaded, bogs typically have few, or even zero, non-native invaders (Zedler and Kercher 2004; Rejmánek *et al*. 2013). Lambdon *et al*. (2008) found that only roughly 10 % of all the non-native plants found in Europe occurred in mires, bogs and fens, making them the least invaded non-marine ecosystems across Europe. Similarly, in a comprehensive analysis of the flora of Catalonia, the Czech Republic and Great Britain, Chytrý *et al*. (2008) found that bogs were among the habitats with the lowest proportion of non-native plants: the percentage of neophytes (plants that arrived after 1500 AD) out of the total flora was zero for Catalonia and the Czech Republic, and only 0.2 % for Great Britain.

Observational evidence suggests that invasions that do occur in bogs can often be attributed to disturbances that ameliorate at least one stressor and/or increase the spread of non-native propagules. Loope *et al*. (1992) reported that in montane bogs in Haleakala National Park, Hawaii, disturbance by feral pigs allowed several invasive plants to displace native plants. Undisturbed bogs were less invaded, and native plants tended to recover in bogs where pigs were excluded. In a tamarack bog in Ohio, USA, hydrologic changes that resulted in an amelioration of a variety of stressors (e.g. increasing nutrient levels and pH) were implicated in the invasion of both native and non-native species (Miletti *et al*. 2005).

While invasions of non-native species into bogs are rare, more common is the encroachment of native vegetation, which is again generally attributed to anthropogenic reductions in stressors. Experimental manipulations often demonstrate the importance of multiple combined stressors in reducing the invasibility of bogs to encroaching natives. For example, in a mesocosm study, Limpens *et al*. (2003) concluded that N deposition improved performance of vascular plants, but that expansion into bogs was still limited by a high water table and low P. Similarly, in a fertilization experiment in a desiccated bog, Tomassen *et al*. (2004) concluded that while N deposition could enhance invasion of a grass—*Molinia caerulea*—invasion of other vascular species would occur only with concurrent increases in P.

### Shaded terrestrial systems

Low-light terrestrial environments such as closed-canopy forests have long been considered resistant to invasion (Baker 1986; Rejmánek 1989; Von Holle *et al*. 2003), and evidence suggests that many shaded ecosystems are indeed less invaded. For instance, forests with closed canopies in California and Europe have lower proportions of non-native species than nearby open habitats (Rejmánek *et al*. 2013). Shaded forest understories often have lower total abundances of non-natives compared with forest edges (e.g. in old-growth forest in Indiana, USA; Brothers and Spingarn 1992), and certain invasive species may only be found in canopy gaps within dense forest (e.g. *Ailanthus altissima* (tree of heaven) in old-growth forest in New York, USA; Knapp and Canham 2000). Additionally, lower percentages of non-native weeds have been found in roadside habitats with closed canopies relative to roadsides with open canopies (Frankel 1977; Parendes and Jones 2000).

Low light is an abiotic stress that is usually directly related to a biotic cause: the shading species. Despite a potentially confounding role of competition, there is compelling evidence that low invadedness of highly shaded habitats may be caused, at least in part, by poorer performance of invasive species relative to natives in low-light conditions, which has been demonstrated in many experiments that manipulate light alone. For example, in Hawaii, experimental reduction of light availability reduced growth and biomass of invasive grasses to a greater extent than native tree and shrub species (Funk and McDaniel 2010). In a review of studies comparing the performance of native to non-native plant species, Daehler (2003) found that native performance equalled or exceeded that of non-natives in low-light conditions in 7 of 10 studies.

The knowledge that invasive species often perform more poorly than natives under greater shade stress has been used as a restoration tool. For example, retaining plantation trees has been used in tropical and subtropical forests to suppress invasive grasses (Parrotta *et al*. 1997; Loh and Daehler 2008). The extent of shading likely determines the chances of invasion success; for instance, establishment of potentially invasive *Miscanthus* species is inhibited only at very low-light levels (West *et al*. 2014).

Despite the general pattern of lower relative performance of invasive species in highly shaded terrestrial environments, a number of studies have shown that certain invasive species outperform natives under all light conditions (Pattison *et al*. 1998; Zheng *et al*. 2009), and that increasing shade does not necessarily reduce invader abundance (Baars and Kelly 1996; Cabin *et al*. 2002). Thus, as in all stressful habitats, invasibility of low-light environments depends largely on the traits of the non-native species (Martin *et al*. 2009; Funk 2013).

### Shaded wetland, riparian and aquatic systems

As in terrestrial systems, low light availability appears to limit invasions of non-native plants in wetland and riparian zones—systems that are otherwise typically highly invaded (Alpert *et al*. 2000; Zedler and Kercher 2004). Canopy gaps and high-light areas created by flooding and sediment deposition are often implicated in the high invasibility of wetlands and riparian zones (Zedler and Kercher 2004; Schnitzler *et al*. 2007), suggesting that light limitation reduces invasibility. Field surveys corroborate this. For example, the presence of an invasive grass, *Glyceria maxima* (reed sweet-grass), along stream banks in Victoria, Australia, decreased with greater riparian overstory cover and was absent in areas of highest riparian shading (Loo *et al*. 2009). Similarly, the invasive grass *Phragmites australis* (common reed) was significantly less likely to be found in roadside drainage ditches in Quebec, Canada when dense woody cover was present (Albert *et al*. 2013). In a meta-analysis of non-native species diversity in European riparian forests, Schnitzler *et al*. (2007) implicated greater light availability as one reason that willow-poplar communities had a higher number and percentage of non-native species than other riparian community types.

Experimental work, though sparse, also points to a limiting effect of light on invasions in wetlands. For example, Maurer and Zedler (2002) found that rhizome establishment and biomass of the invasive *Phalaris arundinacea* (reed canarygrass) were reduced under greater canopy shading under both field and greenhouse conditions in Wisconsin, USA. As in terrestrial environments, shade may give natives a competitive advantage over invasive species in wetlands. In a greenhouse experiment, Chen *et al*. (2013) simulated the effects of different understory light levels on the growth of native and invasive mangroves, finding that while invasive mangroves responded negatively to high levels of shade, native mangroves did not.

Low light may be less important in reducing plant invasions in purely aquatic systems. While light limitation often influences the distribution of submersed macrophytes (Bornette and Puijalon 2011), evidence that light limitation hinders non-native submersed macrophytes more than natives or reduces invasibility is lacking or mixed (e.g. Ali *et al*. 2011). In a test of photosynthetic rates under different light intensities of one non-native and six native submersed macrophytes from Lake George, NY, USA, Madsen *et al*. (1991) classified the non-native *Myriophyllum spicatum* (Eurasian watermilfoil) as high-light adapted and all six natives as ‘shade-tolerant’. However, shading of up to 94 % incident light did not hinder establishment success of invasive *M. spicatum* in artificial stream channels in California, USA (Zefferman 2014).

### High elevations

High-elevation regions are characterized by low temperatures, a short growing/productive period (daily or seasonal), high UV exposure, low available soil nutrients, increased water stress and, in some locations, daily freeze-thaw cycles. These stressors may help explain the observation that high-elevation areas have relatively few non-native species (Alpert *et al*. 2000; Rejmánek *et al*. 2013). Surveys spanning more than 14 locations over seven continents found a general pattern of decreasing numbers of non-native species from low- and mid-elevations to high elevations (Western and Juvik 1983; Pauchard and Alaback 2004; Arévalo *et al*. 2005; Becker *et al*. 2005; Daehler 2005; McDougall *et al*. 2005; Kalwij *et al*. 2008; Pauchard *et al*. 2009; Giraldo-Cañas 2010; Haider *et al*. 2010; Jakobs *et al*. 2010; Alexander *et al*. 2011; Khuroo *et al*. 2011; Marini *et al*. 2012, 2013; but see Paiaro *et al*. 2007, 2011 for counter-examples). Reviews of country- and continent-wide distributions of invasive and non-native species in the Czech Republic, China and North America have also found that mountainous or high-elevation areas have fewer non-native species than lower regions (Baker 1986; Feng and Zhu 2010; Pyšek *et al*. 2012).

It is less clear whether this pattern holds for proportional representation of non-natives within the species pool. Native species richness typically declines at high elevations as well (but see Becker *et al*. 2005, Alexander *et al*. 2011), and studies that report information on native species richness typically do not explicitly report proportional richness. However, non-native richness was found to decline with elevation both absolutely and relative to native plant richness in the Italian Alps (Marini *et al*. 2012, 2013), the Swiss Alps (Becker *et al*. 2005), in woody species of the Kashmir Himalayas (Khuroo *et al*. 2011), in roadside weeds of Hawaiian Islands (Western and Juvik 1983; Jakobs *et al*. 2010), in roadside vegetation of Chile (Alexander *et al*. 2011) and in grasses of the northern Andes >2000 m (Giraldo-Cañas 2010). Similarly, Palmer (2006) found that non-native species richness had a sharper decline with elevation than native species richness in North America. However, in the Canary Islands, studies suggest an absolute but not a relative decline in non-native plant richness at high elevations (Arévalo *et al*. 2005; Alexander *et al*. 2011).

### High latitudes

Like high elevations, high-latitude regions (>50°N or S) exert stresses associated with low temperatures that can prevent or slow the establishment, growth and life-cycle completion of plants. High latitudes also exhibit high seasonal variation in photoperiod, with long periods of low light energy.

Surveys along latitudinal gradients at the scale of continents or countries have shown that non-native species richness decreases above 40°. This decline with latitude has been observed for naturalized species (introduced species that can sustain a population without requiring repeated reintroductions, *sensu* Richardson *et al*. 2000) in all biomes and vegetation community types in continental Europe (Sax 2001), Chile (Fuentes *et al*. 2013) and for all non-native plants in the contiguous USA (Stohlgren *et al*. 2005). In riparian forests in Europe, latitudes above >50°N were host to the fewest non-natives Schnitzler *et al*. (2007). Because native species richness also decreases with increasing latitude, it remains unclear whether the proportion of non-native to native species typically declines with latitude in these cases. However, for North American flora, Palmer (2006) found that non-native plant richness is more negatively affected by latitude than native richness.

The Sub-Antarctic and Antarctic regions also show a general pattern of decreasing non-native plant richness with higher latitude, but not necessarily relative to native richness. In a survey of 25 Sub-Antarctic Islands, densities of non-native species per island (richness/area) decreased with latitude of the island, but proportion of non-native to total richness did not (Chown *et al*. 1998). The Antarctic continent only has two naturalized vascular plants and these are restricted to the milder maritime region (Frenot *et al*. 2005), but Antarctica has only two native vascular plants (Robinson *et al*. 2003).

For the Arctic, it has been noted that naturalized non-natives represent a low to null percentage of species in regional floras (Elven 2011), but research has not explicitly compared this with proportions of non-natives in adjacent lower-latitude areas. Hotspots of non-native richness and abundance in areas of high human activity do exist. For example, areas around settlements on the Arctic archipelago of Svalbard (74–81°N) have 28–37 non-native species present (native species: 165), but it is unclear how many of these are sustained only because of continuous reintroductions or conditions maintained by humans (Ware *et al*. 2012).

Experiments, though rare, suggest that the harsh conditions imposed by high elevations and latitudes could be responsible for low invasibility. For example, it has been shown with lab simulations of polar temperatures and field sowing experiments at polar latitudes (>60°) that vascular plant species introduced to the Arctic or Antarctic (whether from temperate or sub-polar regions) often either do not germinate, or fail to reproduce and form a sustainable population (Smith 1996; Ware *et al*. 2012). Furthermore, winter snowpack depth has been shown to limit the establishment and population growth rates of two invasive shrubs at higher elevations in the Sierra Nevada mountains of California (Stevens and Latimer 2015).

## Hypotheses

**Figure 1.**
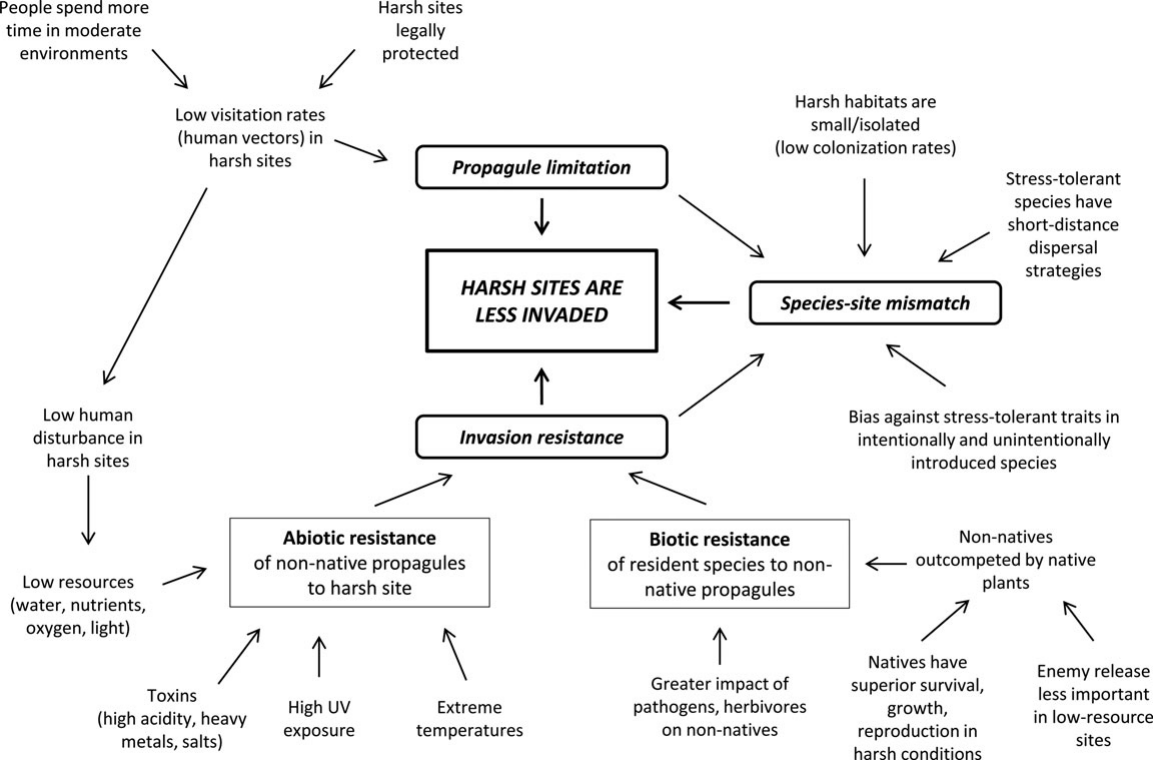
Framework of hypotheses for why harsh sites are less invaded.

The preceding sections provide considerable evidence that abiotically stressful sites are less invaded than more moderate environments [summarized in **Supporting Information**]. Although exceptions occur, this pattern holds across multiple dimensions of plant stress. However, potential explanations of this phenomenon include more than abiotic stress alone, because successful invasion is ultimately influenced not only by the abiotic environment, but also by species traits and propagule pressure (Lonsdale 1999; Theoharides and Dukes 2007). Here we propose a framework for explaining the pattern of low invadedness of harsh sites (Fig. 1). We divide explanations for this pattern into two broad classes. *Propagule limitation mechanisms* assume that non-native species are less likely to be transported to, or disperse among, harsh sites. *Invasion resistance mechanisms* assume that potential invaders are not necessarily dispersal-limited but instead limited by the abiotic stressors of the harsh site or by interactions with resident species. In reality, these classes of mechanisms occupy two ends of a continuum in which dispersal and site characteristics are both important, and *hybrid mechanisms* occur when propagule limitation and invasion resistance mechanisms operate in concert. These mechanisms are relevant to the invasibility of any ecosystem, but here we discuss them in the context of harsh sites specifically.

Two lines of empirical evidence can help distinguish propagule limitation mechanisms from invasion resistance mechanisms in harsh sites. First, the observation of increased invasion through time without an accompanying reduction in a stressor suggests that propagule limitation may have been at least partially responsible for initially low levels of invasion. Second, if experimental amelioration of a stressor results in greater success of invaders without an accompanying increase in propagule pressure, it suggests that the stressor is indeed a direct or indirect cause of invasion resistance. Many of the studies we reviewed imply invasion resistance by showing increased invader abundance or competitive performance following reduction of stressors. However, studies that simply correlate richness or cover of invaders with metrics of harshness are generally not able to distinguish the relative importance of propagule limitation vs invasion resistance. In the following sections, we discuss in greater detail the differences in the evidence for, and underlying causes of, propagule limitation and invasion resistance as drivers of low invadedness of harsh sites.

### Propagule limitation mechanism

The inverse relationship between plant invasion and harshness could be driven by greater propagule limitation at harsh sites compared with more moderate sites. This could include low numbers of propagules arriving at one time, and/or low frequencies of introductions (Simberloff 2009). The propagule limitation mechanism allows for the possibility that any non-native species could invade harsh sites if introduced in sufficient numbers. For instance, low numbers of non-native propagules reaching harsh sites are often implicated in low levels of invasion, without the assumption that the propagules belong to stress-tolerant species (Lonsdale 1999; Alpert *et al*. 2000; Rejmánek *et al*. 2013).

Harsh sites may experience particularly low propagule pressure of non-native species because people (i.e. potential vectors) may visit harsh habitats at lower rates (Alpert *et al*. 2000) due to cultural/aesthetic preferences or practical or institutional limitations. Harsh sites may be more likely than moderate sites to be designated as reserves, either because they are rare or because they are less useful for development (Scott *et al*. 2001), thus reducing human intrusion. Evidence exists for reduced human impacts at a range of harsh sites, including low nutrient and rocky soils (Scott *et al*. 2001; Lavergne *et al*. 2005), and arid, high-latitude and high-elevation regions (Scott *et al*. 2001; Sanderson *et al*. 2002).

Evidence for propagule limitation includes a positive correlation between human use and plant invasion among similarly harsh sites. For example, areas of higher human occupancy or use in high-latitude regions tend to have greater non-native species richness compared with areas of lower human presence at similar latitudes (Chown *et al*. 1998, 2005; Fuentes *et al*. 2013). Pickering *et al*. (2007) found a positive association between tourism infrastructure and presence of non-native species in high-elevation alpine regions of an Australian national park. We do note that sites more visited by humans may also be more disturbed, thus confounding propagule limitation with invasion resistance mechanisms (see below).

Additional evidence to support the propagule limitation mechanism includes successful invasion of cosmopolitan invaders into harsh sites following their introduction (e.g. Alexander *et al*. 2011). For instance, the broadly adapted perennial grasses *Poa annua* and *Poa pratensis* have become invasive in the Antarctic once introduced by humans (Frenot *et al*. 2005; Molina-Montenegro *et al*. 2014).

### Invasion resistance mechanisms

Invasion resistance mechanisms refer to the ecosystem properties that reduce invasibility of a site to non-native species, and we divide these into two main types: *abiotic resistance* and *biotic resistance* (see also Alpert *et al*. 2000; Rejmánek *et al*. 2013). *Abiotic resistance* plays a direct role in reducing the invasibility of a harsh ecosystem, while *biotic resistance* operates indirectly, as mediated by resident species.

#### Abiotic resistance

The abiotic resistance mechanism requires that the degree of physiological stress imposed by a harsh site prevents the establishment or population growth of potential invaders, even in the absence of negative interactions with native species. Potential invaders can be intolerant of many different stressors, including critical resource scarcity (e.g. low nutrients, water, light), environmental conditions that slow or halt metabolism (e.g. temperature extremes, anoxia) or the presence of toxins (e.g. heavy metals, salts) (Table 1; see also review by Alpert *et al*. 2000).

Signals of abiotic resistance involve failure by potential invaders to establish in harsh sites despite the presence of propagules and absence of competitors. Because of the ethical difficulties of experimentally introducing non-native propagules into harsh habitats, however, experimental tests that could definitively exclude propagule limitation as an operative mechanism are rare (but see Ross *et al*. 2008; Stevens and Latimer 2015). Instead, experiments that show increased invasion of harsh sites when stressors are ameliorated are the most common and conclusive evidence for abiotic stress reducing invasibility, particularly when controlling for the level of competition or when conducted in the absence of competition from natives. For example, both field and laboratory soil transplant experiments in the Antarctic have shown that non-native non-vascular plants are already present in the soil propagule bank and will germinate and survive when temperature and moisture are raised (Kennedy 1996). Many studies outlined throughout the ‘Harsh Habitats’ sections above show a positive effect of increases in water, light and nutrients on the richness or cover of invasive species in sites where these resources are limiting.

#### Biotic resistance

Under the biotic resistance mechanism, negative interactions between potential invaders and resident native species reduce invasion risk (Elton 1958; Levine *et al*. 2004). Here we argue that these negative interactions may play a particularly important role in harsh sites. This is because invasive plants commonly have life history traits that are associated with high-resource costs, such as rapid growth rates, high specific leaf area and low water-use efficiency (reviewed in Blumenthal 2006; Rejmánek *et al*. 2013), which may make them especially vulnerable to biotic resistance in harsh sites.

Competition with native plant species (especially in the establishment phase) is one example of biotic resistance. Although competitive interactions are often considered less important in stressful ecosystems (Grime 1977), species competition can determine whether or not coexistence is possible even under harsh conditions (Chesson and Huntly 1997). In harsh sites, resource scarcity may limit the competitive advantage conferred to fast-growing potential invaders, and biotic resistance may become the proximate cause that prevents their invasion. Stress-tolerant natives may outcompete stress-intolerant invaders if invaders need to devote a greater proportion of resources to metabolic maintenance (survival) and therefore have fewer resources available for growth and fecundity (Shea and Chesson 2002). Similarly, if stress-adapted natives are more efficient at assimilating scarce resources, they may drive resources down to levels that are inhospitable for potential invaders.

It can be difficult to disentangle whether potential invaders of harsh sites are physiologically intolerant to abiotic conditions rather than competitively inferior to the native plant community, and in many cases both mechanisms may be in play. The most effective means of distinguishing abiotic and biotic resistance is through controlled experiments that simultaneously manipulate harsh conditions and native abundance. For example, through factorial experiments, Going *et al*. (2009) and Gerhardt and Collinge (2007) showed that abiotic stressors and competitors had additive negative effects on non-natives in serpentine grassland sites and vernal pools, respectively. It is also important to note that in certain cases, native biota may be sources of abiotic stress as well as competitors, thereby muddying the distinction between abiotic and biotic resistance mechanisms. Examples include low light in the understory because of shading by canopy vegetation, and reduced pH in bogs due to excretion of acids by mosses. In a meta-analysis of the effects of biotic resistance on invasive plant species, Levine *et al*. (2004) concluded that competitive exclusion rarely keeps invaders out entirely but often limits their abundance. Therefore, if competitive exclusion is the primary mechanism driving low invasion in a harsh site, we may be more likely to see evidence in terms of low cover rather than low richness of non-natives.

Biotic resistance to non-native plants may also come from resident herbivores and pathogens. Native plants adapted to harsh conditions often have enhanced chemical or structural defences due to the high resource cost of repairing damaged tissue, while high resource-adapted invaders are less likely to be resistant to disease and herbivory (Chapin 1991). Therefore, resident enemies may contribute to the pattern of lower invadedness in harsh sites by suppressing potential invaders, which could lead to competitive exclusion by better-adapted native plants, or to mortality even in the absence of competitors (Shea and Chesson 2002).

Furthermore, while ‘enemy release’ is often cited as a mechanism behind successful invasion of non-native plants (Blossey 2011), enemy release may be less important in sites that are naturally low in resources. Non-native plants that are well adapted to high resource conditions are likely to benefit most from enemy release, because plants with resource-grabbing life history strategies generally allocate energetic resources towards high growth rates rather than defensive chemicals (Blumenthal 2006). Therefore, enemy release may increase the competitive abilities of fast-growing non-natives in high resource sites, but this advantage may be less important in low resource conditions where non-natives are unable to capitalize on available resources (Blumenthal 2006; Blumenthal *et al*. 2009). This potential difference in the effects of enemy release in high and low resource sites may contribute to why harsh sites are relatively less invaded than moderate sites. Experiments that manipulate herbivore or pathogen abundance (e.g. exclusion experiments) on plant communities across a stress gradient would be most useful in distinguishing the role of resident enemies and/or enemy release in contributing to the pattern of low invadedness of harsh sites, but correlative studies can yield insights as well (e.g. Blumenthal *et al*. 2009; Seabloom *et al*. 2009).

### Hybrid mechanism: species–site mismatch

Species–site mismatch is a hybrid mechanism that assumes invasion resistance mechanisms are in effect, but focuses on propagule pressure of species that are ‘well-matched’ to harsh sites. While there may be propagule pressure from non-native species at a harsh site, in a species–site mismatch scenario there is a low probability that these propagules are from species having the necessary resource conservation/acquisition or stress tolerance/avoidance traits required to thrive in a harsh habitat. As a result, harsh sites could be *effectively* propagule-limited, because they are receiving few, if any, propagules that are true potential invaders.

There are many explanations for why harsh sites may receive fewer propagules of well-matched species. Lower human visitation in harsh sites, as discussed above, could result in lower probabilities of transferring well-matched propagules between sites with similar abiotic stressors. Humans may also directly bias propagule pressure: intentionally introduced plants are often chosen for traits—such as fast growth and maturation rates—that make them easy to cultivate in moderate environments where humans are likely to live and work (Colautti *et al*. 2006; Martin *et al*. 2009; Rejmánek *et al*. 2013). Similarly, species that thrive in human-dominated, disturbed landscapes (typically r-selected species) are more likely to be transported unintentionally by humans (Martin *et al*. 2009), while stress-tolerant, specialized plants that could invade harsh environments may be less likely to grow in human-dominated landscapes and therefore less likely to be transported to harsh sites.

Even when non-native stress-tolerant plants are successfully introduced to harsh habitats, their secondary dispersal to other isolated harsh sites may be limited by their dispersal traits. While many invasive plants have long-distance dispersal strategies [e.g. seeds that disperse by wind, water or animal movement (Schupp 2011)], it may be evolutionarily unfavourable for specialized stress-tolerant species to disperse outside of their harsh environments when those habitats are isolated across the landscape (Cousens *et al*. 2008). Even without taking into account differences in dispersal traits, the fact that many harsh sites (e.g. bogs, serpentine soils or alpine sky-islands) are isolated and small in size means that these harsh habitat ‘islands’ may receive fewer naturally dispersing propagules from well-adapted non-native species from similarly harsh and previously invaded sites (*sensu* island biogeography theory, MacArthur and Wilson 1967).

Evidence of the species–site mismatch mechanism comes from an increasing number of studies where harsh environments have become invaded once appropriately matched species have arrived: *Lepidium latifolium* (perennial pepperweed) has become a notorious invader of wetlands and sodic soils (Renz and Blank 2004; Reynolds and Boyer 2010); deserts in the American west have recently been heavily invaded by arid-adapted *Bromus tectorum* (cheatgrass) (Mack 1981), *Brassica tournefortii* (Asian mustard) and *Pennisetum ciliare* (buffelgrass) (Rejmánek *et al*. 2013); serpentine-tolerant ecotypes of *Aegilops triuncialis* (barbed goatgrass) have recently invaded many California serpentine sites (Lyons *et al*. 2010) and introductions of shade-tolerant non-natives have led to invasion of shaded forest understories in both tropical and temperate forests (Sanford *et al*. 2003; Martin and Marks 2006; Martin *et al*. 2009; Holmes *et al*. 2014).

### Disturbance: evidence for invasion resistance mechanisms

The common observation that disturbance facilitates invasion (see review by Hobbs and Huenneke 1992) can be evidence for invasion resistance mechanisms when disturbance ameliorates harsh conditions. When resource limitation hinders potential invaders, disturbance can increase resource availability directly, alleviating abiotic resistance and/or reduce resource uptake by the native plant community, alleviating biotic resistance (Davis *et al*. 2000). Disturbances that facilitate invasions in harsh sites include soil disturbances by animals, such as feral pigs in Montane Hawaiian bogs (Loope *et al*. 1992), and gophers in serpentine grasslands (Koide *et al*. 1987; Hobbs and Mooney 1991). Physical disturbances like treefalls and logging in low-light forests (Rejmánek 1989; Fine 2002; Burnham and Lee 2010) and flood scouring on periodically inundated stream banks (Barden 1987) can also increase recruitment and growth of invasives. When toxins are the dominant stressors in harsh habitats, disturbance may increase invasion in a system by removing toxins from the system. For example, shoreline development reduced salinity (and increased N) in New England salt marshes, leading to increased invasion by non-native *Phragmites australis* (Silliman and Bertness 2004). When a disturbance constitutes the dominant stressor, such as inundation, modification of existing disturbance regimes may increase invasion of the habitat (e.g. stabilization of river flow regimes in Australia favouring non-native species; Catford *et al*. 2011).

### Caveats and counter-examples

While the majority of evidence we found supports the general pattern of low invadedness of harsh sites, there are an increasing number of studies documenting the ability of non-native species to invade harsh sites, albeit slowly in many cases (Williamson and Harrison 2002; Gerhardt and Collinge 2003; Zedler and Black 2004; Martin *et al*. 2009; Holmes *et al*. 2014). Furthermore, while we have provided many examples that support the hypothesis that native species are superior competitors in harsh sites, this pattern is not consistent (see Daehler 2003 for a review). In fact, in a meta-analysis, Van Kleunen *et al*. (2010) concluded that environmental stress did *not* have a significant effect on the relative performance of natives vs. invasives. We have provided many such counter-examples throughout this review, but these should not be considered a comprehensive list.

In certain cases, invasive plants may perform better than natives in harsh environments when the invaders *themselves* increase abiotic harshness in ways that favour conspecific recruitment. For example, Reinhart *et al*. (2006) found that increased shading from the invasive riparian tree *Acer platanoides* (Norway maple) suppressed native but not conspecific seedlings, and invasive species that increase soil salinity, such as *Tamarix* (saltcedar) species in the western USA (Glenn *et al*. 1998; Ladenburger *et al*. 2006) and *Mesembryanthemum crystallinum* (iceplant) in Mediterranean desert (El-Ghareeb 1991) can lower the relative establishment or growth rates of native species.

Furthermore, there are increasing published examples of populations of non-native species that may have evolved to *become* tolerant of greater stress. Examples include saline-/alkali-tolerant ecotypes of *Festuca perennis* (ryegrass; Dawson *et al*. 2007), cold-tolerant ecotypes of *Echinochloa crus-galli* (barnyard grass) in high latitudes (Roy *et al*. 2000), salt-tolerant ecotypes of *B. tectorum* (Scott *et al*. 2010) in deserts and heavy-metal-tolerant ecotypes of the grasses *Anthoxanthum odoratum* and *Agrostis tenuis* invading mine tailings (McNeilly 1968; Antonovics and Bradshaw 1970; Antonovics 2006). However, for the first three examples, it is not clear whether tolerance evolved after invasion, or if stress-tolerant genotypes arrived later. If the latter, these examples may more appropriately relate to the hypothesis of species-site mismatch (see above), but in the context of well-suited genotypes rather than species.

## Conclusions

Invasion of harsh environments by non-native plant species involves many, often interacting, factors. Overall, the descriptive and experimental evidence from a variety of ecosystems summarized in this paper show a pattern of lower plant invadedness in harsh sites. The extent to which this pattern will persist is unclear—in many systems it already appears to be breaking down, as non-native species continue to spread through human activities, expand their invasive ranges and evolve tolerance to local conditions. As some harsh sites are ameliorated by climate change, N deposition, eutrophication and other anthropogenic changes, they can be expected to become increasingly invaded.

Conservationists and restorationists have long taken encouragement from the fact that edaphically severe sites are often less invaded, thus becoming refuges for native endemic plant species. Understanding the mechanisms behind why harsh sites are less invaded can help managers determine appropriate actions to protect or restore these sites. To the extent that harshness of a site *per se* makes it resistant to invasion, restoration techniques that reinforce the role of the stressor may be effective. For example, soil impoverishment in eutrophied areas (Morgan 1994; Paschke *et al*. 2000; James *et al*. 2011; Török *et al*. 2014) and salt applications in salt marshes with anthropogenic freshwater inflows (Kuhn and Zedler 1997; Uyeda *et al*. 2013) have been used to shift plant communities away from invasive species to native species. However, if propagule limitation is the operative mechanism, management should be more directed towards identifying those species that may be able to successfully invade, or are already beginning to invade, and aggressively intervening to prevent their arrival or catch them in the early stages of invasion. This could include developing early detection and rapid response (EDRR) programmes, and increasing educational outreach and public awareness. Of course, addressing issues of propagule pressure and stress amelioration simultaneously are likely to be most effective at reducing invasions in any harsh site.

## Sources of Funding

This work was supported by fellowships from the University of California, Davis Ecology Graduate Group and Plant Sciences Department, the latter funded by the MacDonald Endowment. This material is based upon work supported by the National Science Foundation Graduate Research Fellowship Program under Grant No. 1148897, to G.K.C., L.V.M., D.J.N.Y., and T.E. Additional funding came from the National Science Foundation: DEB 10-50543 to T.P.Y.

## Contributions by the Authors

T.P.Y. conceived of this review paper. Each author wrote at least one section of the manuscript. E.Z. integrated these into a coherent manuscript, with help from J.T.S. All authors contributed edits.

## Conflict of Interest Statement

None declared.

## Supporting Information

The following additional information is available in the online version of this article –

**Table S1.** Summary of literature listed in this paper that provide observational evidence that harsh sites are less invaded and experimental evidence of invasion resistance. Full citations are listed in the ‘Literature cited’ of the main text.

## References

[PLV056C1] Abraham JK, Corbin JD, D'Antonio CM 2009 California native and exotic perennial grasses differ in their response to soil nitrogen, exotic annual grass density, and order of emergence. Plant Ecology 201:445–456. 10.1007/s11258-008-9467-1

[PLV056C2] Albert A, Brisson J, Dubé J, Lavoie C 2013 Do woody plants prevent the establishment of common reed along highways? Insights from southern Quebec. Invasive Plant Science and Management 6:585–592. 10.1614/IPSM-D-13-00025.1

[PLV056C3] Alexander JM, Kueffer C, Daehler CC, Edwards PJ, Pauchard A, Seipel T, MIREN Consortium. 2011 Assembly of nonnative floras along elevational gradients explained by directional ecological filtering. Proceedings of the National Academy of Sciences of the USA 108:656–661. 10.1073/pnas.1013136108 21187380 PMC3021079

[PLV056C4] Ali MM, Hassan SA, Shaheen ASM 2011 Impact of riparian trees shade on aquatic plant abundance in conservation islands. Acta Botanica Croatica 70:245–258.

[PLV056C5] Alpert P, Bone E, Holzapfel C 2000 Invasiveness, invasibility and the role of environmental stress in the spread of non-native plants. Perspectives in Plant Ecology, Evolution and Systematics 3:52–66. 10.1078/1433-8319-00004

[PLV056C6] Antonovics J 2006 Evolution in closely adjacent plant populations X: long-term persistence of prereproductive isolation at a mine boundary. Heredity 97:33–37. 10.1038/sj.hdy.6800835 16639420

[PLV056C7] Antonovics J, Bradshaw AD 1970 Evolution in closely adjacent plant populations. VIII. Clinal patterns at a mine boundary. Heredity 25:349–362. 10.1038/hdy.1970.36 16639420

[PLV056C8] Arévalo JR, Delgado JD, Otto R, Naranjo A, Salas M, Fernández-Palacios JM 2005 Distribution of alien vs. native plant species in roadside communities along an altitudinal gradient in Tenerife and Gran Canaria (Canary Islands). Perspectives in Plant Ecology, Evolution and Systematics 7:185–202. 10.1016/j.ppees.2005.09.003

[PLV056C9] Atkinson D 1973 Some general effects of phosphorus deficiency on growth and development. New Phytologist 72:101–111. 10.1111/j.1469-8137.1973.tb02014.x

[PLV056C10] Baars R, Kelly D 1996 Survival and growth responses of native and introduced vines in New Zealand to light availability. New Zealand Journal of Botany 34:389–400. 10.1080/0028825X.1996.10410702

[PLV056C11] Baker HG 1986 Patterns of plant invasion in North America. In: Mooney HA, Drake JA, eds. Ecology of biological invasions of North America and Hawaii. New York: Springer, 44–57.

[PLV056C12] Bangerth F 1979 Calcium-related physiological disorders of plants. Annual Review of Phytopathology 17:97–122. 10.1146/annurev.py.17.090179.000525

[PLV056C13] Barden LS 1987 Invasion of *Microstegium vimineum* (Poaceae), an exotic, annual, shade-tolerant, C_4_ grass, into a North Carolina floodplain. American Midland Naturalist 118:40–45. 10.2307/2425626

[PLV056C14] Bashkin M, Stohlgren TJ, Otsuki Y, Lee M, Evangelista P, Belnap J 2003 Soil characteristics and plant exotic species invasions in the Grand Staircase—Escalante National Monument, Utah, USA. Applied Soil Ecology 22:67–77. 10.1016/S0929-1393(02)00108-7

[PLV056C15] Becker T, Dietz H, Billeter R, Buschmann H, Edwards PJ 2005 Altitudinal distribution of alien plant species in the Swiss Alps. Perspectives in Plant Ecology, Evolution and Systematics 7:173–183. 10.1016/j.ppees.2005.09.006

[PLV056C16] Begna SH, Dwyer LM, Cloutier D, Assemat L, DiTommaso A, Zhou X, Prithiviraj B, Smith DL 2002 Decoupling of light intensity effects on the growth and development of C_3_ and C_4_ weed species through sucrose supplementation. Journal of Experimental Botany 53:1935–1940. 10.1093/jxb/erf043 12177133

[PLV056C17] Belnap J, Phillips SL 2001 Soil biota in an ungrazed grassland: response to annual grass (*Bromus tectorum*) invasion. Ecological Applications 11:1261–1275. 10.1890/1051-0761(2001)011[1261:SBIAUG:2.0.CO;2

[PLV056C18] Bernstein L 1975 Effects of salinity and sodicity on plant growth. Annual Review of Phytopathology 13:295–312. 10.1146/annurev.py.13.090175.001455

[PLV056C19] Blossey B 2011 Enemy release hypothesis. In: Simberloff D, Rejmánek M, eds. Encyclopedia of biological invasions. Berkeley, CA: University of California Press, 193–196.

[PLV056C20] Blumenthal DM 2006 Interactions between resource availability and enemy release in plant invasion. Ecology Letters 9:887–895. 10.1111/j.1461-0248.2006.00934.x 16796578

[PLV056C21] Blumenthal DM, Mitchell CE, Pyšek P, Jarošik V 2009 Synergy between pathogen release and resource availability in plant invasion. Proceedings of the National Academy of Sciences of the USA 106:7899–7904. 10.1073/pnas.0812607106 19416888 PMC2674393

[PLV056C22] Bornette G, Puijalon S 2011 Response of aquatic plants to abiotic factors: a review. Aquatic Sciences 73:1–14. 10.1007/s00027-010-0162-7

[PLV056C23] Boughton EH, Quintana-Ascencio PF, Nickerson D, Bohlen PJ 2011 Management intensity affects the relationship between non-native and native species in subtropical wetlands. Applied Vegetation Science 14:210–220. 10.1111/j.1654-109X.2010.01116.x

[PLV056C24] Brooks ML 2003 Effects of increased soil nitrogen on the dominance of alien annual plants in the Mojave Desert. Journal of Applied Ecology 40:344–353. 10.1046/j.1365-2664.2003.00789.x

[PLV056C25] Brooks ML 2009 Spatial and temporal distribution of non-native plants in upland areas of the Mojave Desert. In: Webb RH, Fenstermaker LF, Heaton JS, Hughson DL, McDonald EV, Miller DM, eds. The Mojave Desert: ecosystem processes and sustainability. Reno: University of Nevada Press, 101–124.

[PLV056C26] Brothers TS, Spingarn A 1992 Forest fragmentation and alien plant invasion of central Indiana old-growth forests. Conservation Biology 6:91–100. 10.1046/j.1523-1739.1992.610091.x

[PLV056C27] Buisson E, Anderson S, Holl KD, Corcket E, Hayes GF, Peeters A, Dutoit T 2008 Reintroduction of *Nassella pulchra* to California coastal grasslands: effects of topsoil removal, plant neighbour removal and grazing. Applied Vegetation Science 11:195–204. 10.3170/2008-7-18357

[PLV056C28] Burgess TL, Bowers JE, Turner RM 1991 Exotic plants at the desert laboratory, Tucson, Arizona. Madroño 38:254–265.

[PLV056C29] Burke IC, Bontti EE, Barrett JE, Lowe PN, Lauenroth WK, Riggle R 2013 Impact of labile and recalcitrant carbon treatments on available nitrogen and plant communities in a semiarid ecosystem. Ecological Applications 23:537–545. 10.1890/12-0015.1 23734484

[PLV056C30] Burnham KM, Lee TD 2010 Canopy gaps facilitate establishment, growth, and reproduction of invasive *Frangula alnus* in a *Tsuga canadensis* dominated forest. Biological Invasions 12:1509–1520. 10.1007/s10530-009-9563-8

[PLV056C31] Cabin RJ, Weller SG, Lorence DH, Cordell S, Hadway LJ, Montgomery R, Goo D, Urakami A 2002 Effects of light, alien grass, and native species additions on Hawaiian dry forest restoration. Ecological Applications 12:1595–1610. 10.1890/1051-0761(2002)012[1595:EOLAGA:2.0.CO;2

[PLV056C32] Caldwell MM, Björn LO, Bornman JF, Flint SD, Kulandaivelu G, Teramura AH, Tevini M 1998 Effects of increased solar ultraviolet radiation on terrestrial ecosystems. Journal of Photochemistry and Photobiology B: Biology 46:40–52. 10.1016/S1011-1344(98)00184-5 12659537

[PLV056C33] Catford JA, Downes BJ, Gippel CJ, Vesk PA 2011 Flow regulation reduces native plant cover and facilitates exotic invasion in riparian wetlands. Journal of Applied Ecology 48:432–442. 10.1111/j.1365-2664.2010.01945.x

[PLV056C34] Chapin FS III 1991 Integrated responses of plants to stress: a centralized system of physiological responses. BioScience 41:29–36. 10.2307/1311538

[PLV056C35] Chapin FS III, Bloom AJ, Field CB, Waring RH 1987 Plant responses to multiple environmental factors. BioScience 37:49–57. 10.2307/1310177

[PLV056C36] Chase JM, Knight TM 2006 Effects of eutrophication and snails on Eurasian watermilfoil (*Myriophyllum spicatum*) invasion. Biological Invasions 8:1643–1649. 10.1007/s10530-005-3933-7

[PLV056C37] Chen L, Peng S, Li J, Lin Z, Zeng Y 2013 Competitive control of an exotic mangrove species: restoration of native mangrove forests by altering light availability. Restoration Ecology 21:215–223. 10.1111/j.1526-100X.2012.00892.x

[PLV056C38] Cherwin KL, Seastedt TR, Suding KN 2009 Effects of nutrient manipulations and grass removal on cover, species composition, and invasibility of a novel grassland in Colorado. Restoration Ecology 17:818–826. 10.1111/j.1526-100X.2008.00418.x

[PLV056C39] Chesson P, Huntly N 1997 The roles of harsh and fluctuating conditions in the dynamics of ecological communities. The American Naturalist 150:519–553. 10.1086/286080 18811299

[PLV056C40] Chiarucci A, Baker AJM 2007 Advances in the ecology of serpentine soils. Plant and Soil 293:1–2. 10.1007/s11104-007-9268-7

[PLV056C41] Chown SL, Gremmen NJM, Gaston KJ 1998 Ecological biogeography of southern ocean islands: species-area relationships, human impacts, and conservation. The American Naturalist 152:562–575. 10.1086/286190 18811364

[PLV056C42] Chown SL, Hull B, Gaston KJ 2005 Human impacts, energy availability and invasion across Southern Ocean Islands. Global Ecology and Biogeography 14:521–528. 10.1111/j.1466-822x.2005.00173.x

[PLV056C43] Chytrý M, Maskell LC, Pino J, Pyšek P, Vilà M, Font X, Smart SM 2008 Habitat invasions by alien plants: a quantitative comparison among Mediterranean, subcontinental and oceanic regions of Europe. Journal of Applied Ecology 45:448–458. 10.1111/j.1365-2664.2007.01398.x

[PLV056C44] Colautti RI, Grigorovich IA, MacIsaac HJ 2006 Propagule pressure: a null model for biological invasions. Biological Invasions 8:1023–1037. 10.1007/s10530-005-3735-y

[PLV056C45] Collinge SK, Ray C, Gerhardt F 2011 Long-term dynamics of biotic and abiotic resistance to exotic species invasion in restored vernal pool plant communities. Ecological Applications 21:2105–2118. 10.1890/10-1094.1 21939047

[PLV056C46] Corbin JD, D'Antonio CM, Bainbridge SJ 2004 Tipping the balance in restoration of native plants. In: Gordon MS, Bartol SM, eds. Experimental approaches to conservation biology. Berkeley, CA: University of California Press, 154–179.

[PLV056C47] Cousens R, Dytham C, Law R 2008 *Dispersal**in plants: a population perspective*. Oxford: Oxford University Press, 159–160.

[PLV056C48] Cronk QC, Fuller JL 1995 Plant invaders: the threat to natural ecosystems. Berlin: Springer.

[PLV056C49] Daehler CC 2003 Performance comparisons of co-occurring native and alien invasive plants: implications for conservation and restoration. Annual Review of Ecology, Evolution, and Systematics 34:183–211. 10.1146/annurev.ecolsys.34.011802.132403

[PLV056C50] Daehler CC 2005 Upper-montane plant invasions in the Hawaiian Islands: patterns and opportunities. Perspectives in Plant Ecology, Evolution and Systematics 7:203–216. 10.1016/j.ppees.2005.08.002

[PLV056C51] Daehler CC, Strong DR 1996 Status, prediction and prevention of introduced cordgrass *Spartina* spp. invasions in Pacific estuaries, USA. Biological Conservation 78:51–58. 10.1016/0006-3207(96)00017-1

[PLV056C52] Davis MA, Grime JP, Thompson K 2000 Fluctuating resources in plant communities: a general theory of invasibility. Journal of Ecology 88:528–534. 10.1046/j.1365-2745.2000.00473.x

[PLV056C53] Dawson K, Veblen KE, Young TP 2007 Experimental evidence for an alkali ecotype of *Lolium multiflorum*, an exotic invasive annual grass in the Central Valley, CA, USA. Biological Invasions 9:327–334. 10.1007/s10530-006-9036-2

[PLV056C54] Dimitrakopoulos PG, Galanidis A, Siamantziouras A-SD, Troumbis AY 2005 Short-term invasibility patterns in burnt and unburnt experimental Mediterranean grassland communities of varying diversities. Oecologia 143:428–437. 10.1007/s00442-004-1808-8 15711823

[PLV056C55] Dyer AR, Rice KJ 1999 Effects of competition on resource availability and growth of a California bunchgrass. Ecology 80:2697–2710. 10.1890/0012-9658(1999)080[2697:EOCORA:2.0.CO;2

[PLV056C56] El-Ghareeb R 1991 Vegetation and soil changes induced by *Mesembryanthemum crystallinum* L. in a mediterranean desert ecosystem. Journal of Arid Environments 20:321–330.

[PLV056C57] Elton CS 1958 The ecology of invasions by animals and plants. London: Methuen, 181 pp.

[PLV056C58b] Elven R, Murray DF, Razzhivin V, Yurtsev BA 2011 Checklist of the panarctic flora (PAF). Oslo, Norway: CAFF/University of Oslo.

[PLV056C58a] Emam T 2015 The role of soil biota, abiotic stress, and provenance in plant interactions and restoration. Dissertation, University of California, Davis, CA.

[PLV056C58] Engelhardt KAM 2011 Aquatic eutrophication. In: Simberloff D, Rejmánek M, eds. Encyclopedia of biological invasions. Berkeley, CA: University of California Press, 209–213.

[PLV056C59] Feng J, Zhu Y 2010 Alien invasive plants in China: risk assessment and spatial patterns. Biodiversity and Conservation 19:3489–3497. 10.1007/s10531-010-9909-7

[PLV056C60] Fine PVA 2002 The invasibility of tropical forests by exotic plants. Journal of Tropical Ecology 18:687–705.

[PLV056C61] Fisher JL, Veneklaas EJ, Lambers H, Loneragan WA 2006 Enhanced soil and leaf nutrient status of a Western Australian Banksia woodland community invaded by *Ehrharta calycina* and *Pelargonium capitatum*. Plant and Soil 284:253–264. 10.1007/s11104-006-0042-z

[PLV056C62] Flores TA, Setterfield SA, Douglas MM 2005 Seedling recruitment of the exotic grass *Andropogon gayanus* (Poaceae) in northern Australia. Australian Journal of Botany 53:243–249. 10.1071/BT03154

[PLV056C63] Foy CD, Chaney RL, White MC 1978 The physiology of metal toxicity in plants. Annual Review of Plant Physiology 29:511–566. 10.1146/annurev.pp.29.060178.002455

[PLV056C64] Frankel RE 1977 Ruderal vegetation along some California roadsides. University of California Publications in Geography 20:1–163.

[PLV056C65] Frenot Y, Chown SL, Whinam J, Selkirk PM, Convey P, Skotnicki M, Bergstrom DM 2005 Biological invasions in the Antarctic: extent, impacts and implications. Biological Reviews 80:45–72. 10.1017/S1464793104006542 15727038

[PLV056C66] Fuentes N, Pauchard A, Sánchez P, Esquivel J, Marticorena A 2013 A new comprehensive database of alien plant species in Chile based on herbarium records. Biological Invasions 15:847–858. 10.1007/s10530-012-0334-6

[PLV056C67] Funk JL 2013 The physiology of invasive plants in low-resource environments. Conservation Physiology 1:1–17. 10.1093/conphys/cot026 PMC4806624 27293610

[PLV056C68] Funk JL, McDaniel S 2010 Altering light availability to restore invaded forest: the predictive role of plant traits. Restoration Ecology 18:865–872. 10.1111/j.1526-100X.2008.00515.x

[PLV056C69] Gerhardt F, Collinge SK 2003 Exotic plant invasions of vernal pools in the Central Valley of California, USA. Journal of Biogeography 30:1043–1052. 10.1046/j.1365-2699.2003.00911.x

[PLV056C70] Gerhardt F, Collinge SK 2007 Abiotic constraints eclipse biotic resistance in determining invasibility along experimental vernal pool gradients. Ecological Applications 17:922–933. 10.1890/05-1146 17494407

[PLV056C71] Gioria M, Osborne BA 2014 Resource competition in plant invasions: emerging patterns and research needs. Frontiers in Plant Science 5:501 10.3389/fpls.2014.00501 25324851 PMC4179379

[PLV056C72] Giraldo-Cañas D 2010 Distribution and invasion of C-3 and C-4 grasses (Poaceae) along an altitudinal gradient in the Andes of Colombia. Caldasia 32:65–86.

[PLV056C73] Glenn E, Tanner R, Mendez S, Kehret T, Moore D, Garcia J, Valdes C 1998 Growth rates, salt tolerance and water use characteristics of native and invasive riparian plants from the delta of the Colorado River, Mexico. Journal of Arid Environments 40:281–294. 10.1006/jare.1998.0443

[PLV056C74] Goergen E, Daehler CC 2001 Reproductive ecology of a native Hawaiian grass (*Heteropogon contortus*; Poaceae) versus its invasive alien competitor (*Pennisetum setaceum*; Poaceae). International Journal of Plant Sciences 162:317–326. 10.1086/319587

[PLV056C75] Going BM, Hillerislambers J, Levine JM 2009 Abiotic and biotic resistance to grass invasion in serpentine annual plant communities. Oecologia 159:839–847. 10.1007/s00442-008-1264-y 19139921

[PLV056C76] Gram WK, Borer ET, Cottingham KL, Seabloom EW, Boucher VL, Goldwasser L, Micheli F, Kendall BE, Burton RS 2004 Distribution of plants in a California serpentine grassland: are rocky hummocks spatial refuges for native species? Plant Ecology 172:159–171. 10.1023/B:VEGE.0000026332.57007.7b

[PLV056C77] Grime JP 1977 Evidence for the existence of three primary strategies in plants and its relevance to ecological and evolutionary theory. The American Naturalist 111:1169–1194. 10.1086/283244

[PLV056C78] Grime JP 1989 The stress debate: symptom of impending synthesis? Biological Journal of the Linnean Society 37:3–17. 10.1111/j.1095-8312.1989.tb02002.x

[PLV056C79] Grime JP, Curtis AV 1976 The interaction of drought and mineral nutrient stress in calcareous grassland. Journal of Ecology 64:975–988. 10.2307/2258819

[PLV056C80] Gu B 2006 Environmental conditions and phosphorus removal in Florida lakes and wetlands inhabited by *Hydrilla verticillata* (Royle): implications for invasive species management. Biological Invasions 8:1569–1578. 10.1007/s10530-005-5851-0

[PLV056C81] Haider S, Alexander J, Dietz H, Trepi L, Edwards PJ, Kueffer C 2010 The role of bioclimatic origin, residence time and habitat context in shaping non-native plant distributions along an altitudinal gradient. Biological Invasions 12:4003–4018. 10.1007/s10530-010-9815-7

[PLV056C82] Harrington GN 1991 Effects of soil moisture on shrub seedling survival in semi-arid grassland. Ecology 72:1138–1149. 10.2307/1940611

[PLV056C83] Harrison S 1999a Local and regional diversity in a patchy landscape: native, alien, and endemic herbs on serpentine. Ecology 80:70–80. 10.1890/0012-9658(1999)080[0070:LARDIA:2.0.CO;2

[PLV056C84] Harrison S 1999b Native and alien species diversity at the local and regional scales in a grazed California grassland. Oecologia 121:99–106. 10.1007/s004420050910 28307892

[PLV056C85] Haubensak KA, D'Antonio CM 2011 The importance of nitrogen-fixation for an invader of a coastal California grassland. Biological Invasions 13:1275–1282. 10.1007/s10530-010-9904-7

[PLV056C86] He Q, Cui B, An Y 2012 Physical stress, not biotic interactions, preclude an invasive grass from establishing in forb-dominated salt marshes. PLoS ONE 7:e33164 10.1371/journal.pone.0033164 22432003 PMC3303875

[PLV056C87] Hobbs RJ, Huenneke LF 1992 Disturbance, diversity, and invasion: implications for conservation. Conservation Biology 6:324–337. 10.1046/j.1523-1739.1992.06030324.x

[PLV056C88] Hobbs RJ, Mooney HA 1991 Effects of rainfall variability and gopher disturbance on serpentine annual grassland dynamics. Ecology 72:59–68. 10.2307/1938902

[PLV056C89] Hobbs RJ, Gulmon SL, Hobbs VJ, Mooney HA 1988 Effects of fertiliser addition and subsequent gopher disturbance on a serpentine annual grassland community. Oecologia 75:291–295. 10.1007/BF00378612 28310849

[PLV056C90] Holdredge C, Bertness MD, Von Wettberg E, Silliman BR 2010 Nutrient enrichment enhances hidden differences in phenotype to drive a cryptic plant invasion. Oikos 119:1776–1784. 10.1111/j.1600-0706.2010.18647.x

[PLV056C91] Holland RF, Jain S 1988 Vernal pools. In: Barbour MG, Major J, eds. Terrestrial vegetation of California. Sacramento: California Native Plant Society, 515–533.

[PLV056C92] Holmes KA, Greco SE, Berry AM 2014 Pattern and process of fig (*Ficus carica*) invasion in a California riparian forest. Invasive Plant Science and Management 7:46–58. 10.1614/IPSM-D-13-00045.1

[PLV056C93] Hopper SD 2002 Weeds on granite outcrops in temperate Australia, South Africa and the USA. 13th Australian Weeds Conference Papers & Proceedings Plant Protection Society of WA, Perth, 96–99.

[PLV056C94] Houle G, Phillips DL 1989 Seasonal variation and annual fluctuation in granite outcrop plant communities. Vegetatio 80:25–35. 10.1007/BF00049138

[PLV056C95] Huenneke LF, Hamburg SP, Koide R, Mooney HA, Vitousek PM 1990 Effects of soil resources on plant invasion and community structure in Californian serpentine grassland. Ecology 71:478–491. 10.2307/1940302

[PLV056C96] Hultine KR, Belnap J, van Riper C III, Ehleringer JR, Dennison PE, Lee ME, Nagler PL, Snyder KA, Uselman SM, West JB 2010 Tamarisk biocontrol in the western United States: ecological and societal implications. Frontiers in Ecology and the Environment 8:467–474. 10.1890/090031

[PLV056C97] Jaffre T 1992 Floristic and ecological diversity of the vegetation on ultramafic rocks in New Caledonia. In: Baker AJM, Proctor J, Reeves RD, eds. The Vegetation of Ultramafic (Serpentine) Soils: Proceedings of the First International Conference on Serpentine Ecology. Intercept, Andover, UK

[PLV056C98] Jakobs G, Kueffer C, Daehler CC 2010 Introduced weed richness across altitudinal gradients in Hawai'i: humps, humans and water-energy dynamics. Biological Invasions 12:4019–4031. 10.1007/s10530-010-9816-6

[PLV056C99] James JJ, Drenovsky RE, Monaco TA, Rinella MJ 2011 Managing soil nitrogen to restore annual grass-infested plant communities: effective strategy or incomplete framework? Ecological Applications 21:490–502. 10.1890/10-0280.1 21563579

[PLV056C100] Kalwij JM, Robertson MP, van Rensburg BJ 2008 Human activity facilitates altitudinal expansion of exotic plants along a road in montane grassland, South Africa. Applied Vegetation Science 11:491–498. 10.3170/2008-7-18555

[PLV056C101] Kennedy AD 1996 Antarctic fellfield response to climate change: a tripartite synthesis of experimental data. Oecologia 107:141–150. 10.1007/BF00327897 28307299

[PLV056C102] Khuroo AA, Weber E, Malik AH, Reshi ZA, Dar GH 2011 Altitudinal distribution patterns of the native and alien woody flora in Kashmir Himalaya, India. Environmental Research 111:967–977. 10.1016/j.envres.2011.05.006 21784423

[PLV056C103] Knapp LB, Canham CD 2000 Invasion of an old-growth forest in New York by *Ailanthus altissima*: sapling growth and recruitment in canopy gaps. Journal of the Torrey Botanical Society 127:307–315. 10.2307/3088649

[PLV056C104] Knapp PA 1996 Cheatgrass (*Bromus tectorum* L) dominance in the Great Basin Desert: history, persistence, and influences to human activities. Global Environmental Change 6:37–52. 10.1016/0959-3780(95)00112-3

[PLV056C105] Koide RT, Huenneke LF, Mooney HA 1987 Gopher mound soil reduces growth and affects ion uptake of two annual grassland species. Oecologia 72:284–290. 10.1007/BF00379280 28311552

[PLV056C106] Kolb A, Alpert P, Enters D, Holzapfel C 2002 Patterns of invasion within a grassland community. Journal of Ecology 90:871–881. 10.1046/j.1365-2745.2002.00719.x

[PLV056C107] Konisky RA, Burdick DM 2004 Effects of stressors on invasive and halophytic plants of New England salt marshes: a framework for predicting response to tidal restoration. Wetlands 24:434–447. 10.1672/0277-5212(2004)024[0434:EOSOIA:2.0.CO;2

[PLV056C108] Kruckeberg AR 1954 The ecology of serpentine soils: a symposium. III. Plant species in relation to serpentine soils. Ecology 35:267–274.

[PLV056C109] Kruckeberg AR 1984 California serpentines: flora, vegetation, soils, and management problems. Berkeley, CA: University of California Press, 180.

[PLV056C110] Kueffer C, Klingler G, Zirfass K, Schumacher E, Edwards PJ, Güsewell S 2008 Invasive trees show only weak potential to impact nutrient dynamics in phosphorus-poor tropical forests in the Seychelles. Functional Ecology 22:359–366. 10.1111/j.1365-2435.2007.01373.x

[PLV056C111] Kuhn NL, Zedler JB 1997 Differential effects of salinity and soil saturation on native and exotic plants of a coastal salt marsh. Estuaries 20:391–403. 10.2307/1352352

[PLV056C112] Ladenburger CG, Hild AL, Kazmer DJ, Munn LC 2006 Soil salinity patterns in *Tamarix* invasions in the Bighorn Basin, Wyoming, USA. Journal of Arid Environments 65:111–128. 10.1016/j.jaridenv.2005.07.004

[PLV056C113] Lake JC, Leishman MR 2004 Invasion success of exotic plants in natural ecosystems: the role of disturbance, plant attributes and freedom from herbivores. Biological Conservation 117:215–226. 10.1016/S0006-3207(03)00294-5

[PLV056C114] Lambdon PW, Pyšek P, Basnou C, Hejda M, Arianoutsou M, Essl F, Jarosik V, Pergl J, Winter M, Anastasiu P, Andriopoulos P, Bazos I, Brundu G, Celesti-Grapow L, Chassot P, Delipetrou P, Josefsson M, Kark S, Klotz S, Kokkoris Y, Kuehn I, Marchante H, Perglova I, Pino J, Vila M, Zikos A, Roy D, Hulme PE 2008 Alien flora of Europe: species diversity, temporal trends, geographical patterns and research needs. Preslia 80:101–149.

[PLV056C115] Larson DL, Anderson PJ, Newton W 2001 Alien plant invasion in mixed-grass prairie: effects of vegetation type and anthropogenic disturbance. Ecological Applications 11:128–141. 10.1890/1051-0761(2001)011[0128:APIIMG:2.0.CO;2

[PLV056C116] Lavergne S, Thuiller W, Molina J, Debussche M 2005 Environmental and human factors influencing rare plant local occurrence, extinction and persistence: a 115-year study in the Mediterranean region. Journal of Biogeography 32:799–811. 10.1111/j.1365-2699.2005.01207.x

[PLV056C117] Lee MA, Power SA 2013 Direct and indirect effects of roads and road vehicles on the plant community composition of calcareous grasslands. Environmental Pollution 176:106–113. 10.1016/j.envpol.2013.01.018 23416745

[PLV056C118] Leishman MR, Thomson VP 2005 Experimental evidence for the effects of additional water, nutrients and physical disturbance on invasive plants in low fertility Hawkesbury Sandstone soils, Sydney, Australia. Journal of Ecology 93:38–49. 10.1111/j.1365-2745.2004.00938.x

[PLV056C119] Leishman MR, Hughes MT, Gore DB 2004 Soil phosphorus enhancement below stormwater outlets in urban bushland: spatial and temporal changes and the relationship with invasive plants. Australian Journal of Soil Research 42:197–202. 10.1071/SR03035

[PLV056C120] Levine JM, Adler PB, Yelenik SG 2004 A meta-analysis of biotic resistance to exotic plant invasions. Ecology Letters 7:975–989. 10.1111/j.1461-0248.2004.00657.x

[PLV056C121] Liancourt P, Viard-Crétat F, Michalet R 2009 Contrasting community responses to fertilization and the role of the competitive ability of dominant species. Journal of Vegetation Science 20:138–147. 10.1111/j.1654-1103.2009.05501.x

[PLV056C122] Limpens J, Berendse F, Klees H 2003 N deposition affects N availability in interstitial water, growth of Sphagnum and invasion of vascular plants in bog vegetation. New Phytologist 157:339–347. 10.1046/j.1469-8137.2003.00667.x 33873643

[PLV056C123] Lockwood JL, Cassey P, Blackburn T 2005 The role of propagule pressure in explaining species invasions. Trends in Ecology and Evolution 20:223–228. 10.1016/j.tree.2005.02.004 16701373

[PLV056C124] Loh RK, Daehler CC 2008 Influence of woody invader control methods and seed availability on native and invasive species establishment in a Hawaiian forest. Biological Invasions 10:805–819. 10.1007/s10530-008-9237-y

[PLV056C125] Lonsdale WM 1999 Global patterns of plant invasions and the concept of invasibility. Ecology 80:1522–1536. 10.1890/0012-9658(1999)080[1522:GPOPIA:2.0.CO;2

[PLV056C126] Loo SE, Mac Nally R, O'Dowd DJ, Lake PS 2009 Secondary invasions: implications of riparian restoration for in-stream invasion by an aquatic grass. Restoration Ecology 17:378–385. 10.1111/j.1526-100X.2008.00378.x

[PLV056C127] Loope LL, Sanchez PG, Tarr PW, Loope WL, Anderson RL 1988 Biological invasions of arid land nature reserves. Biological Conservation 44:95–118. 10.1016/0006-3207(88)90006-7

[PLV056C128] Loope LL, Nagata RJ, Medeiros A 1992 Alien plants in Haleakala National Park. In: Stone CP, Smith CW, Tunison T, eds. Alien plant invasions in native ecosystems of Hawaii. Honolulu: University of Hawaii Cooperative National Park Resources Studies Unit, 551–576.

[PLV056C129] Lortie CJ, Cushman JH 2007 Effects of a directional abiotic gradient on plant community dynamics and invasion in a coastal dune system. Journal of Ecology 95:468–481. 10.1111/j.1365-2745.2007.01231.x

[PLV056C130] Lyons KG, Shapiro AM, Schwartz MW 2010 Distribution and ecotypic variation of the invasive annual barb goatgrass (*Aegilops triuncialis*) on serpentine soil. Invasive Plant Science and Management 3:376–389. 10.1614/IPSM-09-036.1

[PLV056C131] MacArthur RH, Wilson EO 1967 The theory of island biogeography. Princeton, NJ: Princeton University Press.

[PLV056C132] MacDougall AS, Boucher J, Turkington R, Bradfield GE 2006 Patterns of plant invasion along an environmental stress gradient. Journal of Vegetation Science 17:47–56. 10.1111/j.1654-1103.2006.tb02422.x

[PLV056C133] Mack RN 1981 Invasion of *Bromus tectorum* L. into western North America: an ecological chronicle. Agro-Ecosystems 7:145–165. 10.1016/0304-3746(81)90027-5

[PLV056C134] Madsen JD, Hartleb CF, Boylen CW 1991 Photosynthetic characteristics of *Myriophyllum spicatum* and six submersed aquatic macrophyte species native to Lake George, New York. Freshwater Biology 26:233–240. 10.1111/j.1365-2427.1991.tb01732.x

[PLV056C135] Marini L, Battisti A, Bona E, Federici G, Martini F, Pautasso M, Hulme PE 2012 Alien and native plant life-forms respond differently to human and climate pressures. Global Ecology and Biogeography 21:534–544. 10.1111/j.1466-8238.2011.00702.x

[PLV056C136] Marini L, Bertolli A, Bona E, Federici G, Martini F, Prosser F, Bommarc R 2013 Beta-diversity patterns elucidate mechanisms of alien plant invasion in mountains. Global Ecology and Biogeography 22:450–460. 10.1111/geb.12006

[PLV056C137] Maron JL, Connors PG 1996 A native nitrogen-fixing shrub facilitates weed invasion. Oecologia 105:302–312. 10.1007/BF00328732 28307102

[PLV056C138] Maron JL, Marler M 2008 Field-based competitive impacts between invaders and natives at varying resource supply. Journal of Ecology 96:1187–1197. 10.1111/j.1365-2745.2008.01440.x

[PLV056C139] Marschner H 1991 Mechanisms of adaptation of plants to acid soils. Plant and Soil 134:1–20.

[PLV056C140] Martin PH, Marks PL 2006 Intact forests provide only weak resistance to a shade-tolerant invasive Norway maple (*Acer platanoides* L.). Journal of Ecology 94:1070–1079. 10.1111/j.1365-2745.2006.01159.x

[PLV056C141] Martin PH, Canham CD, Marks PL 2009 Why forests appear resistant to exotic plant invasions: intentional introductions, stand dynamics, and the role of shade tolerance. Frontiers in Ecology and the Environment 7:142–149. 10.1890/070096

[PLV056C142] Maurer D, Zedler J 2002 Differential invasion of a wetland grass explained by tests of nutrients and light availability on establishment and clonal growth. Oecologia 131:279–288. 10.1007/s00442-002-0886-8 28547696

[PLV056C143] McDougall KL, Morgan JW, Walsh NG, Williams RJ 2005 Plant invasions in treeless vegetation of the Australian Alps. Perspectives in Plant Ecology, Evolution and Systematics 7:159–171. 10.1016/j.ppees.2005.09.001

[PLV056C144] McLendon T, Redente EF 1991 Nitrogen and phosphorus effects on secondary succession dynamics on a semi-arid sagebrush site. Ecology 72:2016–2024. 10.2307/1941556

[PLV056C145] McNaughton SJ 1968 Structure and function in California grasslands. Ecology 49:962–972. 10.2307/1936547

[PLV056C146] McNeilly T 1968 Evolution in closely adjacent plant populations. III. *Agrostis tenuis* on a small copper mine. Heredity 23:99–108. 10.1038/hdy.1968.8

[PLV056C147] Mengel K, Kosegarten H, Kirkby EA, Appel T 2001 *Principles**of plant nutrition*, 5th edn Dordrecht, The Netherlands: Kluwer Academic Publishers.

[PLV056C148] Mesléard F, Ham LT, Boy V, van Wijck C, Grillas P 1993 Competition between an introduced and an indigenous species: the case of *Paspalum paspalodes* (Michx) Schribner and *Aeluropus littoralis* (Gouan) in the Camargue (southern France). Oecologia 94:204–209. 10.1007/BF00341318 28314033

[PLV056C149] Milchunas DG, Lauenroth WK 1995 Inertia in plant community structure: state changes after cessation of nutrient-enrichment stress. Ecological Applications 5:452–458. 10.2307/1942035

[PLV056C150] Miletti TE, Carlyle CN, Picard CR, Mulac KM, Landaw A, Fraser LH 2005 Hydrology, water chemistry, and vegetation characteristics of a tamarack bog in Bath Township, Ohio: towards restoration and enhancement. Ohio Journal of Science 105:21–30.

[PLV056C151] Miller ME, Belnap J, Beatty SW, Reynolds RL 2006 Performance of *Bromus tectorum* L. in relation to soil properties, water additions, and chemical amendments in calcareous soils of southeastern Utah, USA. Plant and Soil 288:1–18. 10.1007/s11104-006-0058-4

[PLV056C152] Mills JE, Reinartz JA, Meyer GA, Young EB 2009 Exotic shrub invasion in an undisturbed wetland has little community-level effect over a 15-year period. Biological Invasions 11:1803–1820. 10.1007/s10530-008-9359-2

[PLV056C153] Molina-Montenegro MA, Carrasco-Urra F, Acuña-Rodríguez I, Oses R, Torres-Díaz C, Chwedorzewska KJ 2014 Assessing the importance of human activities for the establishment of the invasive *Poa annua* in Antarctica. Polar Research 33:article 21425 10.3402/polar.v33.21425

[PLV056C154] Morgan JP 1994 Soil impoverishment: a little-known technique holds potential for establishing prairie. Restoration and Management Notes 12:55–56.

[PLV056C155] Morgan JW 1998 Patterns of invasion of an urban remnant of a species-rich grassland in southeastern Australia by non-native plant species. Journal of Vegetation Science 9:181–190. 10.2307/3237117

[PLV056C156] Nightingale GT 1948 The nitrogen nutrition of green plants. II. Botanical Review 14:185–221. 10.1007/BF02861554

[PLV056C157] O'Dell RE, Claassen VP 2006 Relative performance of native and exotic grass species in response to amendment of drastically disturbed serpentine substrates. Journal of Applied Ecology 43:898–908. 10.1111/j.1365-2664.2006.01193.x

[PLV056C158] Orcutt DM, Nilsen ET 2000 *The physiology**of plants under stress, volume 2: soil and biotic factors*. New York: John Wiley and Sons.

[PLV056C159] Osakabe Y, Osakabe K, Shinozaki K, Tran LP 2014 Response of plants to water stress. Frontiers in Plant Science 5:1–8. 10.3389/fpls.2014.00086 PMC3952189 24659993

[PLV056C160] Paiaro V, Mangeaud A, Pucheta E 2007 Alien seedling recruitment as a response to altitude and soil disturbance in the mountain grasslands of central Argentina. Plant Ecology 193:279–291. 10.1007/s11258-007-9265-1

[PLV056C161] Paiaro V, Cabido M, Pucheta E 2011 Altitudinal distribution of native and alien plant species in roadside communities from central Argentina. Austral Ecology 36:176–184. 10.1111/j.1442-9993.2010.02134.x

[PLV056C162a] Palmer MW 2006 Scale dependence of native and alien species richness in North American floras. Preslia 78:427–436.

[PLV056C162] Parendes LA, Jones JA 2000 Role of light availability and dispersal in exotic plant invasion along roads and streams in the H. J. Andrews Experimental Forest, Oregon. Conservation Biology 14:64–75. 10.1046/j.1523-1739.2000.99089.x

[PLV056C163] Parida AK, Das AB 2005 Salt tolerance and salinity effects on plants: a review. Ecotoxicology and Environmental Safety 60:324–349. 10.1016/j.ecoenv.2004.06.010 15590011

[PLV056C164] Parrotta JA, Turnbull JW, Jones N, eds. 1997 Catalyzing native forest regeneration on degraded tropical lands. Forest Ecology and Management 99:1–7. 10.1016/S0378-1127(97)00190-4

[PLV056C165] Paschke MW, McLendon T, Redente EF 2000 Nitrogen availability and old-field succession in a shortgrass steppe. Ecosystems 3:144–158. 10.1007/s100210000016

[PLV056C166] Pattison RR, Goldstein G, Ares A 1998 Growth, biomass allocation and photosynthesis of invasive and native Hawaiian rainforest species. Oecologia 117:449–459. 10.1007/s004420050680 28307669

[PLV056C167] Pauchard A, Alaback PB 2004 Influence of elevation, land use, and landscape context on patterns of alien plant invasions along roadsides in protected areas of south-central Chile. Conservation Biology 18:238–248. 10.1111/j.1523-1739.2004.00300.x

[PLV056C168] Pauchard A, Kueffer C, Dietz H, Daehler CC, Alexander J, Edwards PJ, Arévalo JR, Cavieres LA, Guisan A, Haider S, Jakobs G, McDougall K, Millar CI, Naylor BJ, Parks CG, Rew LJ, Seipel T 2009 Ain't no mountain high enough: plant invasions reaching new elevations. Frontiers in Ecology and the Environment 7:479–486. 10.1890/080072

[PLV056C169] Pearce RS 2001 Plant freezing and damage. Annals of Botany 87:417–424. 10.1006/anbo.2000.1352

[PLV056C170a] Perry LG, Blumenthal DM, Monaco TA, Paschke MW, Redente EF 2010 Immobilizing nitrogen to control plant invasion. Oecologia 163:13–14. 20387033 10.1007/s00442-010-1580-x

[PLV056C170] Pickering CM, Bear R, Hill W 2007 Indirect impacts of nature based tourism and recreation: the association between infrastructure and the diversity of exotic plants in Kosciuszko National Park, Australia. Journal of Ecotourism 6:146–157. 10.2167/joe162.0

[PLV056C171] Porembski S 2000 The invasibility of tropical granite outcrops (“inselbergs”) by exotic weeds. Journal of the Royal Society of Western Australia 83:131–137.

[PLV056C172] Prober SM, Thiele KR, Lunt ID, Koen TB 2005 Restoring ecological function in temperate grassy woodlands: manipulating soil nutrients, exotic annuals and native perennial grasses through carbon supplements and spring burns. Journal of Applied Ecology 42:1073–1085. 10.1111/j.1365-2664.2005.01095.x

[PLV056C173] Pyšek P, Chytry M, Pergl J, Sádlo J, Wild J 2012 Plant invasions in the Czech Republic: current state, introduction dynamics, invasive species and invaded habitats. Preslia 84:575–629.

[PLV056C174] Qadir M, Schubert S, Ghafoor A, Murtaza G 2001 Amelioration strategies for sodic soils: a review. Land Degradation and Development 12:357–386. 10.1002/ldr.458

[PLV056C175] Raskin I, Nanda Kumar PBA, Dushenkov S, Salt DE 1994 Bioconcentration of heavy metals by plants. Current Opinion in Biotechnology 5:285–290. 10.1016/0958-1669(94)90030-2

[PLV056C176] Reever Morghan KJ, Seastedt TR 1999 Effects of soil nitrogen reduction on nonnative plants in restored grasslands. Restoration Ecology 7:51–55. 10.1046/j.1526-100X.1999.07106.x

[PLV056C177] Reinhart KO, Gurnee J, Tirado R, Callaway RM 2006 Invasion through quantitative effects: intense shade drives native decline and invasive success. Ecological Applications 16:1821–1831. 10.1890/1051-0761(2006)016[1821:ITQEIS:2.0.CO;2 17069374

[PLV056C178] Rejmánek M 1989 Invasibility of plant communities. In: Drake JA, Mooney HA, di Castri F, Groves R, Kruger F, Rejmánek M, Williamson M, eds. Biological invasions: a global perspective. Chichester: Wiley, 369–388.

[PLV056C179] Rejmánek M, Richardson DM, Pyšek P 2013 Plant invasions and invasibility of plant communities. In: van der Maarel E, Franklin J, eds. Vegetation ecology. Oxford: Blackwell, 332–355.

[PLV056C180] Renz MJ, Blank RR 2004 Influence of Perennial Pepperweed (*Lepidium latifolium*) biology and plant–soil relationships on management and restoration. Weed Technology 18:1359–1363. 10.1614/0890-037X(2004)018[1359:IOPPLL:2.0.CO;2

[PLV056C181] Reynolds LK, Boyer KE 2010 Perennial Pepperweed (*Lepidium latifolium*): properties of invaded tidal marshes. Invasive Plant Science and Management 3:130–138. 10.1614/IPSM-D-09-00015.1

[PLV056C182] Richardson DM, Pyšek P, Rejmánek M, Barbour MG, Panetta FD, West CJ 2000 Naturalization and invasion of alien plants: concepts and definitions. Diversity and Distributions 6:93–107. 10.1046/j.1472-4642.2000.00083.x

[PLV056C183] Rickey MA, Anderson RC 2004 Effects of nitrogen addition on the invasive grass *Phragmites australis* and a native competitor *Spartina pectinata*. Journal of Applied Ecology 41:888–896. 10.1111/j.0021-8901.2004.00948.x

[PLV056C184] Robinson SA, Wasley J, Tobin AK 2003 Living on the edge—plants and global change in continental and maritime Antarctica. Global Change Biology 9:1681–1717. 10.1046/j.1365-2486.2003.00693.x

[PLV056C185] Ross LC, Lambdon PW, Hulme PE 2008 Disentangling the roles of climate, propagule pressure and land use on the current and potential elevational distribution of the invasive weed *Oxalis pes-caprae* L. on Crete. Perspectives in Plant Ecology, Evolution and Systematics 10:251–258. 10.1016/j.ppees.2008.06.001

[PLV056C186] Roy S, Simon JP, Lapointe FJ 2000 Determination of the origin of the cold-adapted populations of barnyard grass (*Echinochloa crus-galli*) in eastern North America: a total-evidence approach using RAPD DNA and DNA sequences. Canadian Journal of Botany 78:1505–1513.

[PLV056C187] Rozema J, van de Staaij JWM, Tosserams M 1997 Effects of UV-B radiation on plants from agro- and natural ecosystems. In: Lumsden PJ, ed. Plants and UV-B: responses to environmental change. Cambridge: Cambridge University Press, 213–232.

[PLV056C188] Sanderson EW, Jaiteh M, Levy MA, Redford KH, Wannebo AV, Woolmer G 2002 The human footprint and the last of the wild. BioScience 52:891–904. 10.1641/0006-3568(2002)052[0891:THFATL:2.0.CO;2

[PLV056C189] Sanford NL, Harrington RA, Fownes JH 2003 Survival and growth of native and alien woody seedlings in open and understory environments. Forest Ecology and Management 183:377–385. 10.1016/S0378-1127(03)00141-5

[PLV056C190] Sax DF 2001 Latitudinal gradients and geographic ranges of exotic species: implications for biogeography. Journal of Biogeography 28:139–150. 10.1046/j.1365-2699.2001.00536.x

[PLV056C191] Schnitzler A, Hale BW, Alsum EM 2007 Examining native and exotic species diversity in European riparian forests. Biological Conservation 138:146–156. 10.1016/j.biocon.2007.04.010

[PLV056C192] Schooler SS, Cook T, Prichard G, Yeates AG 2010 Disturbance-mediated competition: the interacting roles of inundation regime and mechanical and herbicidal control in determining native and invasive plant abundance. Biological Invasions 12:3289–3298. 10.1007/s10530-010-9722-y

[PLV056C193] Schupp EW 2011 Plant dispersal ability. In: Simberloff D, Rejmánek M, eds. Encyclopedia of biological invasions. Berkeley, CA: University of California Press, 159–165.

[PLV056C194] Scott JM, Davis FW, McGhie RG, Wright RG, Groves C, Estes J 2001 Nature reserves: do they capture the full range of America’s biological diversity? Ecological Applications 11:999–1007. 10.1890/1051-0761(2001)011[0999:NRDTCT:2.0.CO;2

[PLV056C195] Scott JW, Meyer SE, Merrill KR, Anderson VJ 2010 Local population differentiation in *Bromus tectorum* L. in relation to habitat-specific selection regimes. Evolutionary Ecology 24:1061–1080. 10.1007/s10682-010-9352-y

[PLV056C196] Seabloom EW, Harpole WS, Reichman OJ, Tilman D 2003 Invasion, competitive dominance, and resource use by exotic and native California grassland species. Proceedings of the National Academy of Sciences of the USA 100:13384–13389. 10.1073/pnas.1835728100 14595028 PMC263823

[PLV056C197] Seabloom EW, Borer ET, Jolles A, Mitchell CE 2009 Direct and indirect effects of viral pathogens and the environment on invasive grass fecundity in Pacific Coast grasslands. Journal of Ecology 97:1264–1273. 10.1111/j.1365-2745.2009.01550.x

[PLV056C198] Shea K, Chesson P 2002 Community ecology theory as a framework for biological invasions. Trends in Ecology and Evolution 17:170–176. 10.1016/S0169-5347(02)02495-3

[PLV056C199] Silliman BR, Bertness MD 2004 Shoreline development drives invasion of *Phragmites australis* and the loss of plant diversity on New England salt marshes. Conservation Biology 18:1424–1434. 10.1111/j.1523-1739.2004.00112.x

[PLV056C200] Simberloff D 2009 The role of propagule pressure in biological invasions. Annual Review of Ecology, Evolution, and Systematics 40:81–102. 10.1146/annurev.ecolsys.110308.120304

[PLV056C201] Smith RIL 1996 Introduced plants in Antarctica: potential impacts and conservation issues. Biological Conservation 76:135–146. 10.1016/0006-3207(95)00099-2

[PLV056C202] Smith RJ, Fox WT 1973 Soil water and growth of rice and weeds. Weed Science 21:61–63.

[PLV056C203] Steers RJ 2011 Rock outcrops harbor native perennials in type-converted coastal scrub. Western North American Naturalist 70:516–525. 10.3398/064.070.0412

[PLV056C204] Steers RJ, Funk JL, Allen EB 2011 Can resource-use traits predict native vs. exotic plant success in carbon amended soils? Ecological Applications 21:1211–1224. 21774425 10.1890/09-2345.1

[PLV056C205] Stevens JT, Latimer AM 2015 Snowpack, fire, and forest disturbance: interactions affect montane invasions by non-native shrubs. Global Change Biology 21:2379–2393. 25482316 10.1111/gcb.12824

[PLV056C206] Stohlgren TJ, Otsuki Y, Villa CA, Lee M, Belnap J 2001 Patterns of plant invasions: a case example in native species hotspots and rare habitats. Biological Invasions 3:37–50. 10.1023/A:1011451417418

[PLV056C207] Stohlgren TJ, Barnett D, Flather C, Kartesz J, Peterjohn B 2005 Plant species invasions along the latitudinal gradient in the United States. Ecology 86:2298–2309. 10.1890/04-1195

[PLV056C208] Stokes KE 2008 Exotic invasive black willow (*Salix nigra*) in Australia: influence of hydrological regimes on population dynamics. Plant Ecology 197:91–105. 10.1007/s11258-007-9363-0

[PLV056C209] Talluto MV, Suding KN 2008 Historical change in coastal sage scrub in southern California, USA in relation to fire frequency and air pollution. Landscape Ecology 23:803–815. 10.1007/s10980-008-9238-3

[PLV056C210] Tanentzap AJ, Lee WG, Monks A, Ladley K, Johnson PN, Rogers GM, Comrie JM, Clarke DA, Hayman E 2014 Identifying pathways for managing multiple disturbances to limit plant invasions. Journal of Applied Ecology 51:1015–1023. 10.1111/1365-2664.12271

[PLV056C211] Taylor B, Ganf GG 2005 Comparative ecology of two co-occurring floodplain plants: the native *Sporobolus mitchellii* and the exotic *Phyla canescens*. Marine and Freshwater Research 56:431–440. 10.1071/MF04196

[PLV056C212] Theoharides KA, Dukes JS 2007 Plant invasion across space and time: factors affecting nonindigenous species success during four stages of invasion. New Phytologist 176:256–273. 10.1111/j.1469-8137.2007.02207.x 17822399

[PLV056C213] Thomsen MA, D'Antonio CM 2007 Mechanisms of resistance to invasion in a California grassland: the roles of competitor identity, resource availability, and environmental gradients. Oikos 116:17–30. 10.1111/j.2006.0030-1299.14929.x

[PLV056C214] Tomassen HBM, Smolders AJP, Limpens J, Lamers LPM, Roelofs JGM 2004 Expansion of invasive species on ombrotrophic bogs: desiccation or high N deposition? Journal of Applied Ecology 41:139–150. 10.1111/j.1365-2664.2004.00870.x

[PLV056C215] Török K, Szitár K, Halassy M, Szabó R, Szili-Kovács T, Baráth N, Paschke MW 2014 Long-term outcome of nitrogen immobilization to restore endemic sand grassland in Hungary. Journal of Applied Ecology 51:756–765. 10.1111/1365-2664.12220

[PLV056C216] Turitzin SN 1982 Nutrient limitations to plant growth in a California serpentine grassland. American Midland Naturalist 107:95–99. 10.2307/2425191

[PLV056C217] Uyeda KA, Deutschman DH, Crooks JA 2013 Abiotic limitation of non-native plants in the high salt marsh transition zone. Estuaries and Coasts 36:1125–1136. 10.1007/s12237-013-9640-1

[PLV056C218] Van TK, Wheeler GS, Center TD 1999 Competition between *Hydrilla verticillata* and *Vallisneria americana* as influenced by soil fertility. Aquatic Botany 62:225–233. 10.1016/S0304-3770(98)00100-4

[PLV056C219] Van Kleunen M, Weber E, Fischer M 2010 A meta-analysis of trait differences between invasive and non-invasive plant species. Ecology Letters 13:235–245. 10.1111/j.1461-0248.2009.01418.x 20002494

[PLV056C220] Vartapetian BB, Jackson MB 1997 Plant adaptations to anaerobic stress. Annals of Botany 79:3–20. 10.1093/oxfordjournals.aob.a010303

[PLV056C221] Veblen KE, Young TP 2009 A California grasslands alkali specialist, *Hemizonia pungens* ssp*. pungens*, prefers non-alkali soil. Journal of Vegetation Science 20:170–176. 10.1111/j.1654-1103.2009.05537.x

[PLV056C222] Vitousek PM, Howarth RW 1991 Nitrogen limitation on land and in the sea: how can it occur? Biogeochemistry 13:87–115. 10.1007/BF00002772

[PLV056C223] Von Holle B, Delcourt HR, Simberloff D 2003 The importance of biological inertia in plant community resistance to invasion. Journal of Vegetation Science 14:425–432. 10.1111/j.1654-1103.2003.tb02168.x

[PLV056C224] Walker RB 1954 The ecology of serpentine soils: a symposium. II Factors affecting plant growth on serpentine soils. Ecology 35:259–266.

[PLV056C225] Ware C, Bergstrom DM, Müller E, Alsos IG 2012 Humans introduce viable seeds to the Arctic on footwear. Biological Invasions 14:567–577. 10.1007/s10530-011-0098-4

[PLV056C226] Wedin DA, Tilman D 1996 Influence of nitrogen loading and species composition on the carbon balance of grasslands. Science 274:1720–1723. 10.1126/science.274.5293.1720 8939865

[PLV056C227] Weiss SB 1999 Cars, cows, and checkerspot butterflies: nitrogen deposition and management of nutrient-poor grasslands for a threatened species. Conservation Biology 13:1476–1486. 10.1046/j.1523-1739.1999.98468.x

[PLV056C228] Weiss SB 2006 Impacts of nitrogen deposition on California ecosystems and biodiversity: PIER final project report. Sacramento, CA: California Energy Commission.

[PLV056C229] West NM, Matlaga DP, Davis AS 2014 Quantifying targets to manage invasion risk: light gradients dominate the early regeneration niche of naturalized and pre-commercial *Miscanthus* populations. Biological Invasions 16:1991–2001. 10.1007/s10530-014-0643-z

[PLV056C230] Western L, Juvik JO 1983 Roadside plant communities on Mauna Loa, Hawaii. Journal of Biogeography 10:307–316. 10.2307/2844740

[PLV056C231] White TA, Campbell BD, Kemp PD 1997 Invasion of temperate grassland by a subtropical annual grass across an experimental matrix of water stress and disturbance. Journal of Vegetation Science 8:847–854. 10.2307/3237029

[PLV056C232] Whittaker RH 1954 The ecology of serpentine soils. Ecology 35:258–288. 10.2307/1931126

[PLV056C233] Willems JH 2001 Problems, approaches, and results in restoration of Dutch calcareous grassland during the last 30 years. Restoration Ecology 9:147–154. 10.1046/j.1526-100x.2001.009002147.x

[PLV056C234] Williams J, Roberts S, Hill J, Scardaci S, Tibbits G 1990 IPM: managing water for weed control in rice. California Agriculture 44:7–10.

[PLV056C235] Williamson J, Harrison S 2002 Biotic and abiotic limits to the spread of exotic revegetation species. Ecological Applications 12:40–51. 10.1890/1051-0761(2002)012[0040:BAALTT:2.0.CO;2

[PLV056C236] Wilson SD, Gerry AK 1995 Strategies for mixed-grass prairie restoration: herbicide, tilling, and nitrogen manipulation. Restoration Ecology 3:290–298. 10.1111/j.1526-100X.1995.tb00096.x

[PLV056C237] Wiser SK, Buxton RP 2008 Context matters: matrix vegetation influences native and exotic species composition on habitat islands. Ecology 89:380–391. 10.1890/07-0196.1 18409428

[PLV056C238] Wyatt R 1997 Reproductive ecology of granite outcrop plants from the south-eastern United States. Journal of the Royal Society of Western Australia 80:123–129.

[PLV056C239] Xiao-Yun P, Yu-Peng G, Wen-Ju Z, Bo L, Jia-Kuan C 2006 Cover shot and morphological plasticity of invasive *Alternanthera philoxeroides* along a riparian zone in south China*.* Zhiwu Shengtai Xuebao 30:835–843.

[PLV056C240] Xie D, Yu D, Yu L-F, Liu C-H 2010 Asexual propagations of introduced exotic macrophytes *Elodea nuttallii, Myriophyllum aquaticum*, and *M. propinquum* are improved by nutrient-rich sediments in China. Hydrobiologia 655:37–47. 10.1007/s10750-010-0402-9

[PLV056C241] Zedler JB, Kercher S 2004 Causes and consequences of invasive plants in wetlands: opportunities, opportunists, and outcomes. Critical Reviews in Plant Sciences 23:431–452. 10.1080/07352680490514673

[PLV056C242] Zedler JB, Paling E, McComb A 1990 Differential responses to salinity help explain the replacement of native *Juncus kraussii* by *Typha orientalis* in Western Australian salt marshes. Austral Ecology 15:57–72. 10.1111/j.1442-9993.1990.tb01021.x

[PLV056C243] Zedler PH, Black C 2004 Exotic plant invasions in an endemic-rich habitat: the spread of an introduced Australian grass, *Agrostis avenacea* J. F. Gmel., in California vernal pools. Austral Ecology 29:537–546. 10.1111/j.1442-9993.2004.01446.x

[PLV056C244] Zefferman E 2014 Increasing canopy shading reduces growth but not establishment of *Elodea nuttallii* and *Myriophyllum spicatum* in stream channels. Hydrobiologia 734:159–170. 10.1007/s10750-014-1877-6

[PLV056C245] Zheng Y-L, Feng Y-L, Liu W-X, Liao Z-Y 2009 Growth, biomass allocation, morphology, and photosynthesis of invasive *Eupatorium adenophorum* and its native congeners grown at four irradiances. Plant Ecology 203:263–271. 10.1007/s11258-008-9544-5

